# Proteomic analysis of the effect of hemin in breast cancer

**DOI:** 10.1038/s41598-023-35125-4

**Published:** 2023-06-21

**Authors:** G. P. Coló, K. Schweitzer, G. M. Oresti, E. G. Alonso, L. Fernández Chávez, M. Mascaró, G. Giorgi, A. C. Curino, M. M. Facchinetti

**Affiliations:** 1 Laboratorio de Biología del Cáncer, Instituto de Investigaciones Bioquímicas de Bahía Blanca (INIBIBB-UNS-CONICET), Bahía Blanca, Argentina; 2 Laboratorio de Bioquímica de Lípidos, Departamento de Biología, Bioquímica y Farmacia (UNS), Instituto de Investigaciones Bioquímicas de Bahía Blanca (INIBIBB-UNS-CONICET), Argentina, 8000 Bahía Blanca, CP Argentina; 3 Laboratorio de Fisiología Humana, Departamento de Biología, Bioquímica y Farmacia, Universidad Nacional del Sur, San Juan 670, Bahía Blanca, Argentina

**Keywords:** Histocytochemistry, Immunochemistry, Lipids, Proteases, Proteins, Proteomics, Immunoblotting, Immunohistochemistry, Bioinformatics, Immunological techniques, Mass spectrometry, Microscopy, Proteomic analysis, Breast cancer, Cytoskeleton, Cell signalling, RHO signalling, Stress signalling, Iron, Cytoskeletal proteins, GTP-binding protein regulators, Integrins, Metalloproteins, Oncogene proteins, Proteome, Tumour-suppressor proteins, Lipids, Metals, Proteins, Proteomics, Biochemical reaction networks, Cellular signalling networks, Data processing, Functional clustering, Gene ontology, Protein analysis, Proteome informatics, Proteomics, Cancer, Breast cancer, Cancer models, Biochemistry, Biological techniques, Cancer, Cell biology, Chemical biology, Computational biology and bioinformatics, Molecular biology, Diseases, Oncology, Cancer

## Abstract

Heme, an iron-containing prosthetic group found in many proteins, carries out diverse biological functions such as electron transfer, oxygen storage and enzymatic reactions. Hemin, the oxidised form of heme, is used to treat porphyria and also to activate heme-oxygenase (HO) which catalyses the rate-limiting step in heme degradation. Our group has previously demonstrated that hemin displays antitumor activity in breast cancer (BC). The aim of this work has been to study the effect of hemin on protein expression modifications in a BC cell line to gain insight into the molecular mechanisms of hemin antitumor activity. For this purpose, we carried out proteome analysis by Mass Spectrometry (MS) which showed that 1309 proteins were significantly increased in hemin-treated cells, including HO-1 and the proteases that regulate HO-1 function, and 921 proteins were significantly decreased. Furthermore, the MS-data analysis showed that hemin regulates the expression of heme- and iron-related proteins, adhesion and cytoskeletal proteins, cancer signal transduction proteins and enzymes involved in lipid metabolism. By biochemical and cellular studies, we further corroborated the most relevant in-silico results. Altogether, these results show the multiple physiological effects that hemin treatment displays in BC and demonstrate its potential as anticancer agent.

## Introduction

Breast cancer (BC) is a heterogeneous disease which makes the identification of therapeutic strategies particularly challenging. In the clinic, BC is mainly categorised into three subtypes, namely positive estrogen and progesterone hormone receptors, positive epidermal growth factor receptor 2 (HER2) and triple-negative^1^. The first two subtypes are being quite successfully treated through targeted therapy with anti-estrogen and anti-HER2 drugs and monoclonal antibodies. However, the development of resistance to these BC therapies that are initially effective is commonly observed. Moreover, due to the lack of these receptors, hormonal therapy is ineffective on triple-negative BC^2^. Therefore, cytotoxic chemotherapy which is mainly used for the treatment of this cancer type is typically associated with poorer prognosis compared to other BC subtypes^3^. Thus, novel therapeutic options and molecular targets are clearly needed for BC, specifically those BC lacking hormone receptors or being insensitive or resistant to hormone treatment.

Heme (ferroprotoporphyrin IX), an iron-containing prosthetic group found in many cellular proteins, carry out diverse biological functions ranging from oxygen transport, signal transduction, drug metabolism to energy production and microRNA processing^4^. Heme can be either acquired by diet or be synthesised (and metabolised) in the cells. In relation to cancer-related mechanisms, heme has been shown to be involved in cancer initiation and progression by diverse means, namely modulating several energy-related metabolic pathways, altering Tp53 activity and stability, modulating the tumour microenvironment and/or increasing intracellular reactive oxygen species (ROS) concentration^5^. In addition to ROS formation, heme iron is able to generate DNA damage and lipid peroxidation, thus increasing the mutation rate. Therefore, the concentration of heme within cells must be tightly regulated as it can be a cytotoxic compound in high amounts^6^. Cancer cells have been shown to display higher amounts of heme than normal cells, which may be attributed not only to higher synthesis but also to higher import rates^5^. In addition, heme may be degraded by heme oxygenase (HO), thus decreasing the intracellular levels of this molecule. HO is a microsomal enzyme that catalyses the first rate-limiting step in the degradation of heme, yielding equimolar quantities of biliverdin, carbon monoxide and free iron, and thus removing the latter which is a potent pro-oxidant and pro-inflammatory agent^7^. Two different mammalian HO isoforms have been identified: heme oxygenase-1 (HO-1), an inducible 32-kDa isoform which can be induced by various stimuli, such as inflammatory stress, hypoxia and oxidative injury, and HO-2 which is the constitutive isoform^8^. The functions of HO-1 have been attributed not only to its by-products but also to the protein itself acting as a co-transcriptional factor^9^. In addition, it was further known that the enzyme could also modulate several biological processes by regulating the intracellular levels of the pro-oxidant heme^10^.

The oxidised state of heme, named hemin (Fe^3+^ protoporphyrin chloride), is a breakdown product of haemoglobin and has been shown to promote the growth of early hematopoietic progenitors and to be a potent inducer of beta globin gene expression. In addition, it has been shown to exert anti-diabetic and anti-obesity properties in rodents and to induce ferroptosis in platelets^11,12^. Hemin is widely used in the clinic to treat porphyria and is known to increase the expression and activity of HO-1^8,13^. Remarkably, hemin has been demonstrated to display antitumor effects on prostate, breast and colon cancer^14–17^. Previous results from our laboratory showed that treatment of LM3 BC cells with hemin produces a significant impairment in their ability to migrate and invade and also a decrease in their viability. Moreover, hemin treatment of mice bearing LM3-syngeneic tumours produced a decrease in tumour growth and an impairment in the metastatic process^16^. These effects were accompanied by up-regulation of HO-1, which suggests that this enzyme may, at least in part, account for the effects observed. However, HO-independent effects had been previously reported for hemin and heme analogues^18,19^. Having this background in mind, our objective was to study which proteins are modulated downstream of hemin activation of BC cells.

In the last years, there has been an increase in the number of research projects aimed to massively study genomes. Nowadays, the attention is mainly focused on the proteins expressed by these genes, the proteome. In this work we employed mass spectrometry (MS) and bioinformatic analyses to identify the proteins modulated in a BC cell line after hemin treatment and further corroborated some of the most relevant proteins and pathways identified by means of cellular and molecular studies.

## Results

### LM3 cells proteome analysis after hemin treatment

**Figure 1:**
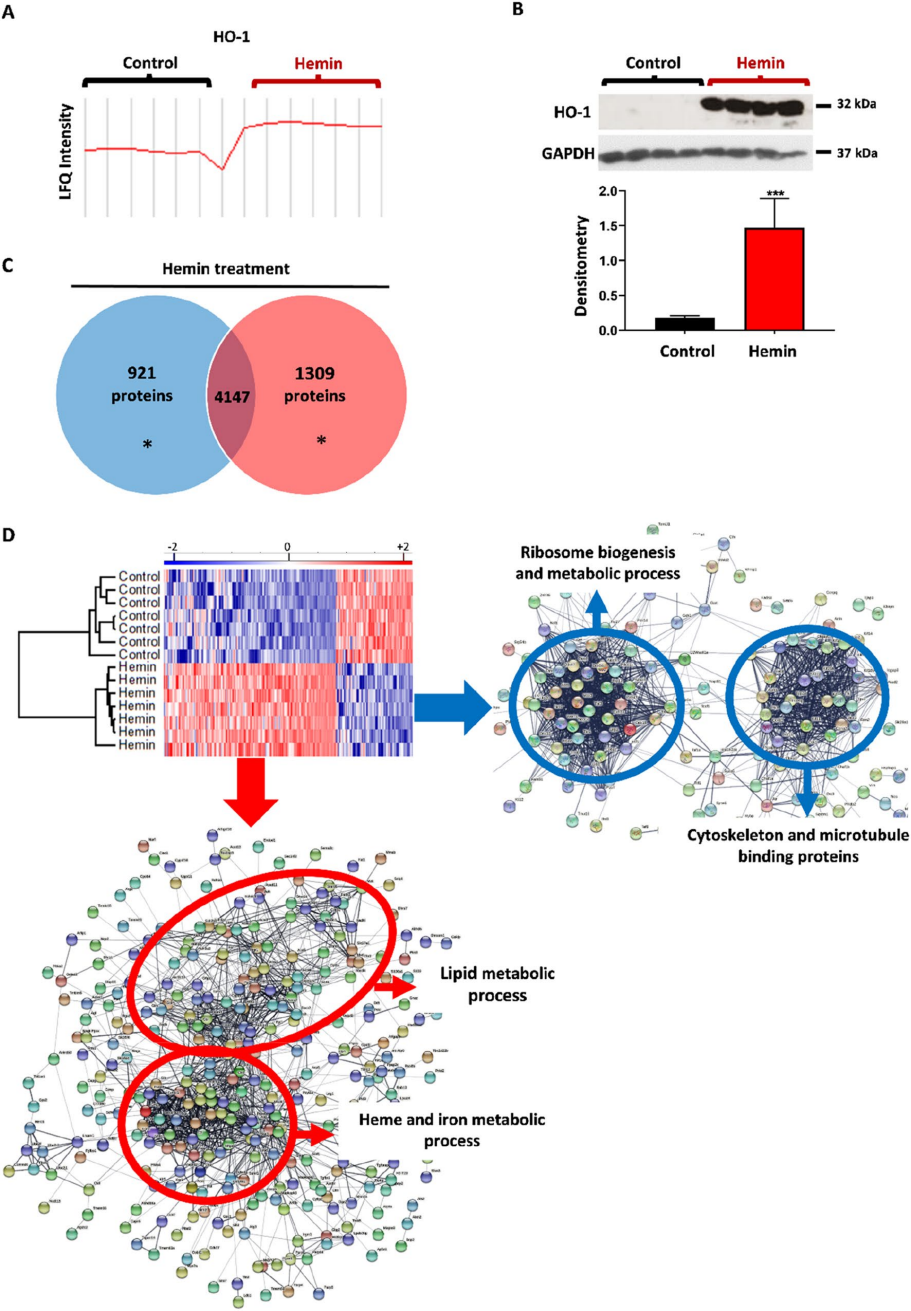
Effects of hemin treatment in LM3 breast cancer cells proteome. (**A**) LM3 cells were treated with 80 µM hemin or vehicle (control) for 24 h. Plot profile of label free quantification (LFQ) intensity of HO-1 protein in control (n = 7) versus hemin treatment (n = 7) is shown. (**B**) Western blot analysis shows the expression of HO-1 protein. GAPDH was used as loading control. Bottom: Bar graph shows the densitometric quantification of HO-1/GAPDH ratio (n = 3) (ImageJ quantification). (**C**) Venn diagram of the total number of proteins differentially expressed with hemin respect to the control. After hemin treatment 1309 proteins were up regulated (red), 921 were down regulated (blue) and 4147 were without any change (*p < 0.05; Student's T-test). (**D**) Heatmap shows the Z-score of significant LM3 protein levels (Student's T-test, S0: 0.5; Permutation-based FDR: 0.05) showing main protein clusters with or without hemin-treatment. The colour code shows in red the up regulated proteins, in blue the down regulated ones and in white the proteins with no significant variation between the vehicle- and hemin-treated cells. Gene ontology-terms enriched in the MS proteome and STRING network analysis showed the clusters of hemin up-modulated proteins (red circles) and the most down-regulated proteins after hemin treatment (blue circles). The functional protein-associated networks show the interconnection between modulated proteins and the edges indicate both functional and physical protein associations.

LM3 cells were treated with hemin (80 µM) or vehicle and the cell lysates were processed for LC–MS/MS peptide detection and MaxQuant protein identification. The hierarchical cluster analysis and the profile plot showed that hemin induced HO-1 expression when compared to vehicle, as expected (Fig. 1A), and this result was corroborated by western blot (control: 0.176 ± 0.035 vs hemin: 1.476 ± 0.385; p = 0.0007) (Fig. 1B). We generated a heatmap with the 6377 proteins detected in seven vehicle- and hemin-treated biological replicates (Supplementary Fig. S1C). Peptides and proteins identified in this study are provided in supplementary Table S1. LC–MS/MS data analysis showed that 6377 out of 6954 peptides changed their expression. Hierarchical cluster analysis of significant proteins identified as differentially expressed between our vehicle- and hemin-treated cells were plotted in a Venn Diagram and heatmap using Perseus software (Fig. 1C,D). We identified 1309 proteins that were significantly increased (red), including HO-1, and 921 proteins that were significantly decreased (blue), in hemin-treated cells with respect to vehicle-treated cells (*p < 0.05; Student's T-test).

**Figure 2:**
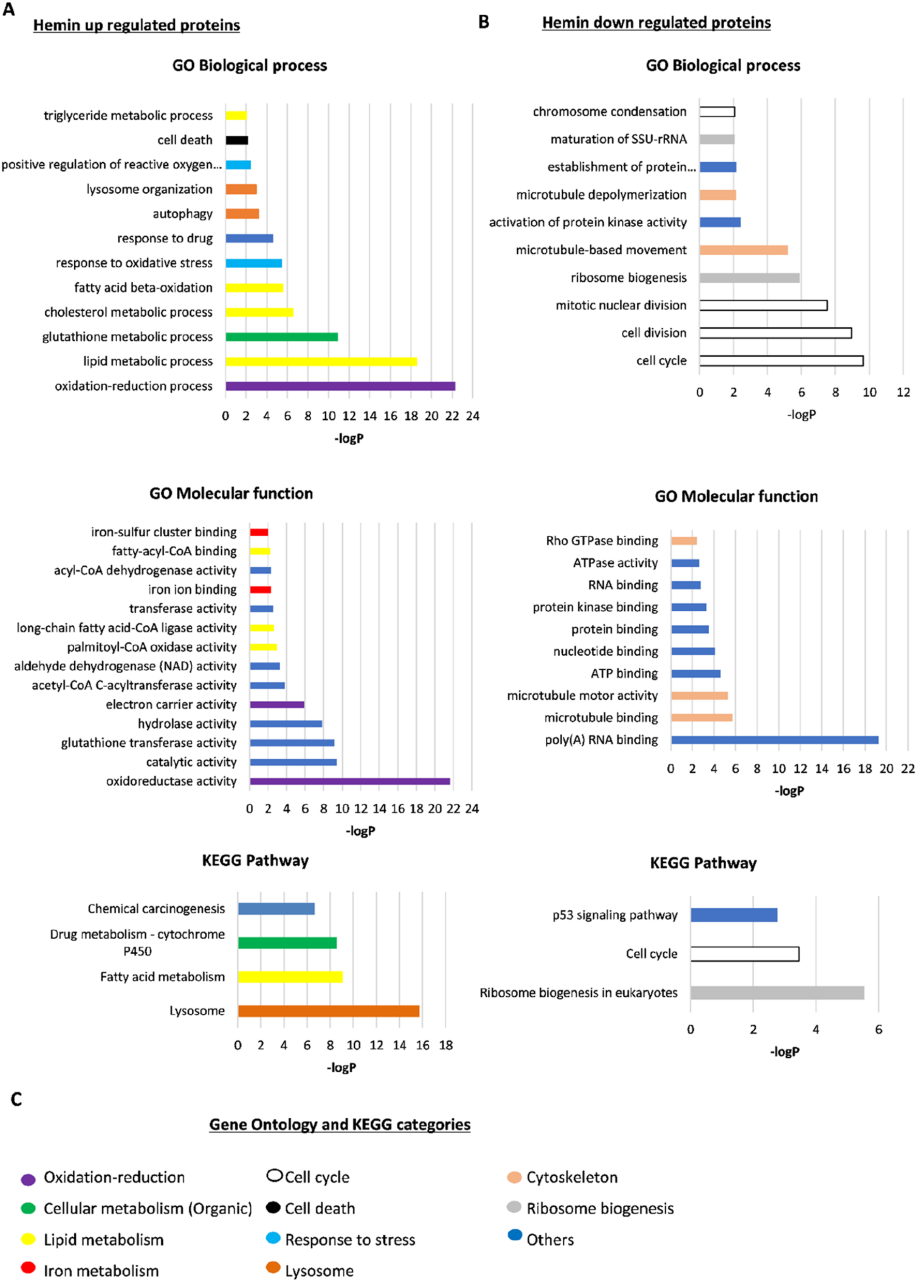
Gene ontology (GO) and Kyoto Encyclopaedia of Genes and Genomes (KEGG) analysis. (**A**) Hemin up-regulated proteins classified according to GO-biological process, GO-molecular function and KEGG dataset pathway. (**B**) Hemin down-regulated proteins classified equally as (**A**). (**C**) Colour code of the eleven global categories for GO-biological process, GO-molecular functions and KEGG annotations.

The Perseus heatmap and STRING network generation with the significant proteins modulated after hemin treatment, revealed a group of upregulated proteins related with lipid and iron metabolism, including HO-1 (Fig. 1D). In addition, within the proteins whose expression decreased after hemin treatment, we noticed two main groups: cytoskeletal proteins and those related to ribosome biogenesis (Fig. 1D). We then performed gene ontology (GO) and Kyoto Encyclopaedia of Genes and Genomes (KEGG) analysis. The GO analysis revealed an enrichment of hemin up-regulated proteins associated with oxidation–reduction, cell death, cellular metabolism, lipid metabolism, lysosome and other functions (Fig. 2A,C). Moreover, the GO analysis for hemin down-regulated proteins showed that those proteins with the lowest expression following hemin treatment were associated with Rho-GTPases and with cell cycle, cytoskeletal dynamics and other processes (Fig. 2B,C). From these up- and down-regulated groups of proteins, we analysed those most relevant for breast cancer progression.

### Hemin treatment modulates the expression of cancer-related proteins

**Figure 3:**
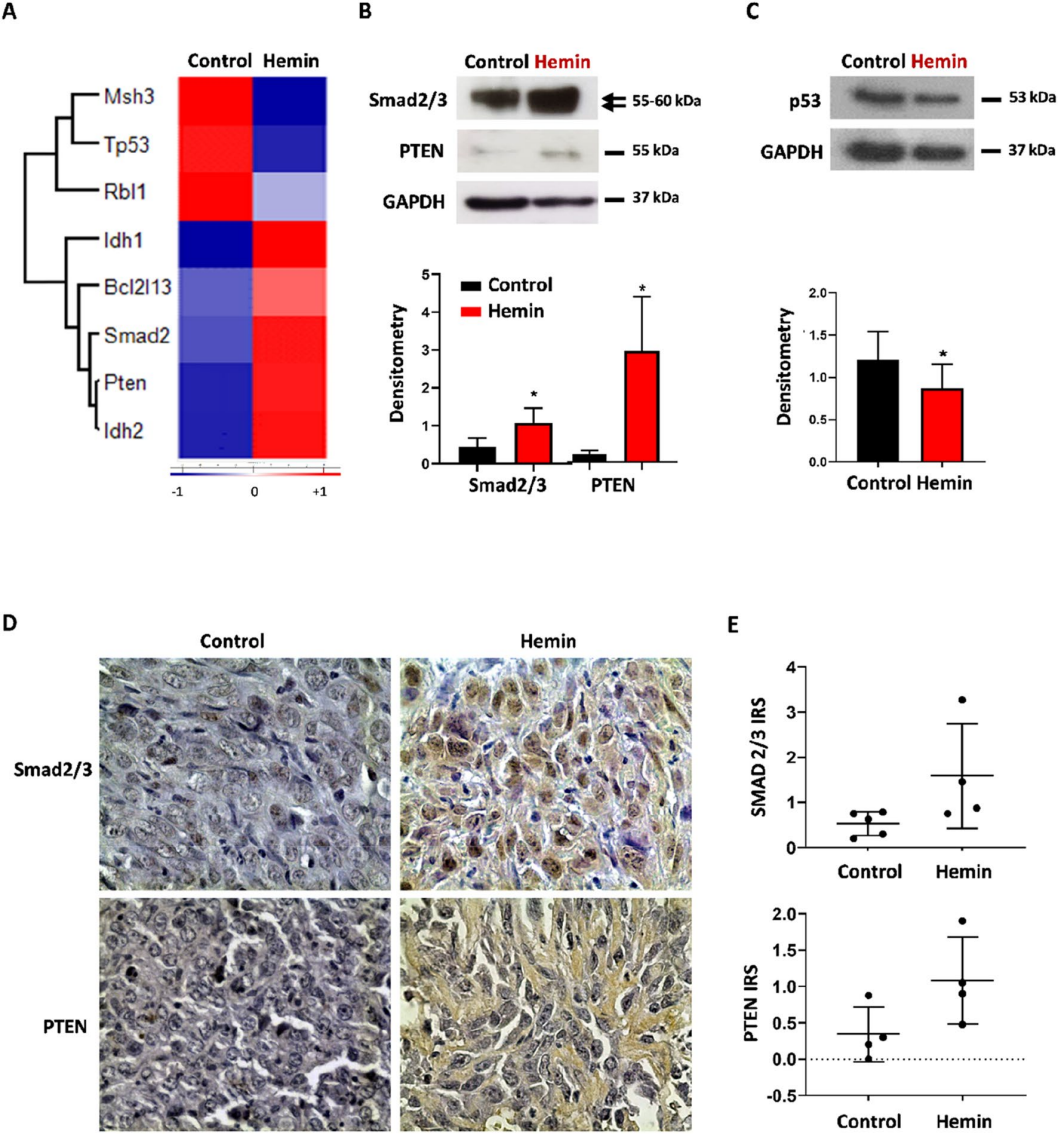
Hemin modulates tumour suppressor proteins. (**A**) Hierarchical cluster analysis shows the significant differences (*p < 0.05; Student's T-test) in the expression of tumour suppressor genes detected in LM3 cells in control- versus hemin-treatment. The Z-score of median MS intensities (n = 7) are colour coded to show relative protein abundance. Shown in red are the up-regulated proteins and shown in blue are the down-regulated proteins after hemin treatment. The bar below indicates how many times the expression increased or decreased (Z-score). Western blot corroborates the protein expression levels of Smad2/3, PTEN (**B**) and p53 (**C**). GAPDH was used as loading control. Bottom: densitometric quantification of each protein (n = 3), (*p < 0.05; Student's T-test). (**D**) Immunohistochemistry of Smad2/3 and PTEN in LM3 syngeneic mouse model biopsies showing the differences in staining between vehicle- and hemin-treated mice. (**E**) Immunoreactive score (IRS) quantification of Smad2/3 and PTEN protein levels (n = 4–5) (*p < 0.05; Student's T-test).

*W*e had previously demonstrated that hemin induces HO-1 expression in the tumour tissues of the LM3 syngeneic mouse model and this result was observed again in this study (Supplementary Fig. S2)^16,51^. In addition, we demonstrated that hemin displays antitumor activity in the breast cancer mouse model, at least in part through HO-1^16^. In agreement with those results, we observed in this study that hemin induces up-regulation of isocitrate dehydrogenase 1 and 2 (Idh1, Idh2), phosphatase and tensin homolog (PTEN) and the signal mediator of the transforming growth factor TGF-β (Smad2) (Fig. 3A). In addition, it produced down-regulation of Tp53, RB transcriptional corepressor like 1 (Rbl1) and MutS Homolog 3 (Msh3) as analysed by MS and bioinformatic analyses (Fig. 3A). We corroborated the results by performing western blot analyses of LM3 lysates for Smad2/3 (control: 0.447 ± 0.23 vs hemin: 1.073 ± 0.480; p = 0.034), PTEN (control: 0.232 ± 0.1 vs hemin: 3.154 ± 1.66; p = 0.03) and p53 (control: 1.207 ± 0.32 vs hemin: 0.870 ± 0.26; p = 0.038) (Fig. 3B,C). The levels of PTEN (control: 0.344 vs hemin: 1.081 IRS; p < 0.05) and Smad2/3 (control: 0.531 vs hemin: 1.583 IRS; p < 0.05) were further corroborated by IHC performed on tumour tissues obtained from the BC animal model (Fig. 3D,E). These studies demonstrate that Smad2/3 and PTEN are upregulated whereas p53 is downregulated in LM3 cells following hemin treatment compared to vehicle treatment.

### Hemin treatment increases the expression of proteases involved in HO-1 C-terminal cleavage

**Figure 4:**
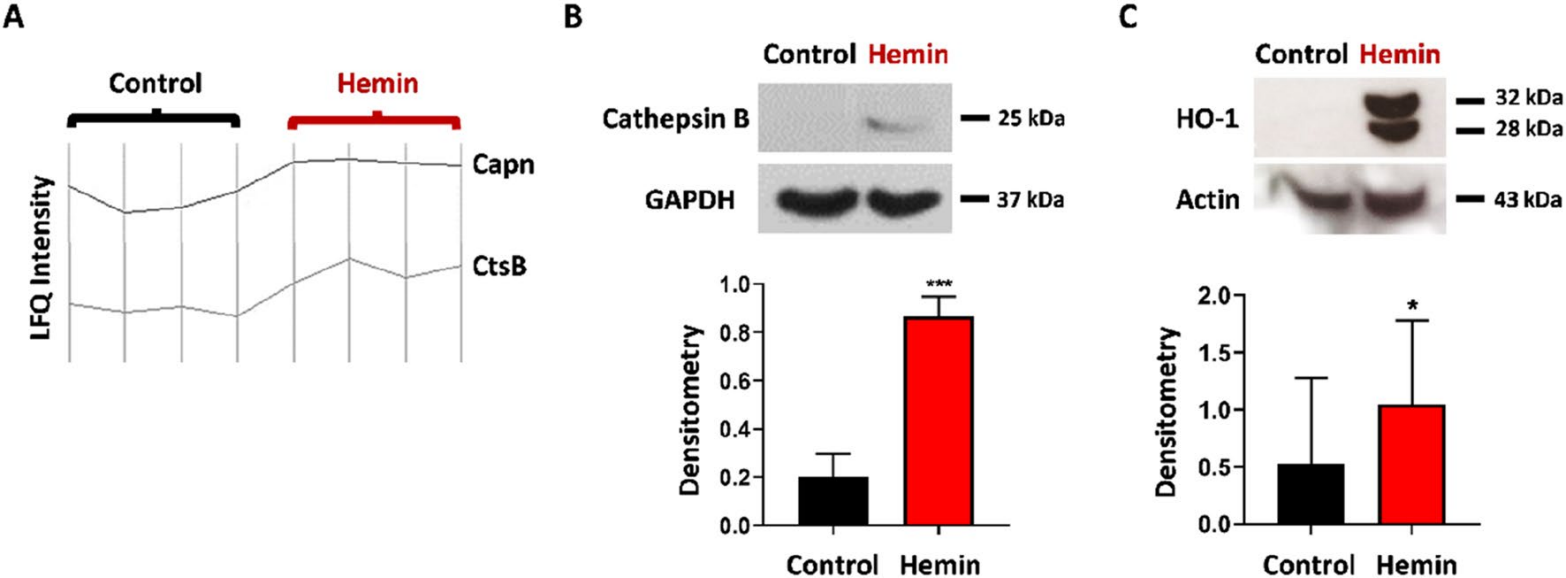
Hemin induces the proteases involved in HO-1 cleavage (**A**) LFQ intensity levels profile of calpain 1 and cathepsin B. (**B**) Western blot shows cathepsin B protein expression. GAPDH was used as loading control. Bottom: Densitometric quantification of HO-1/actin ratio, (***p < 0.001; Student's T-test). (**C**) Western blot shows native (32 kDa) and truncated (28 kDa) HO-1 protein expression after hemin treatment. Actin was used as loading control. Bottom: Densitometric quantification of HO-1/actin ratio (n = 3), (*p < 0.05; Student's T-test).

Recent studies suggest that nuclear HO-1 may have a role in tumour progression independent of its enzymatic activity^20,21^. HO-1 is cleaved before entering the nucleus, generating a 28 kDa C-terminal truncated form. This allows the protein to leave the microsomal membrane to which it is anchored. Proteolytic cleavage of HO-1 C-terminal end involves proteins like calpains, cathepsins and signal peptide peptidase (SPP)^22,23^. Interestingly, these proteases have been linked to breast cancer progression^24,25^. We therefore analysed, by MS, if hemin regulates these proteases in LM3 cells. The LFQ intensity levels profile shows an increase in calpain 1 (Capn) and cathepsin B (CtsB) enzymes in hemin-treated cells with respect to their controls (Fig. 4A). We also demonstrated the upregulation of cathepsin B after hemin treatment of LM3 cells by Western blot (control: 0.201 ± 0.09 vs hemin: 0.867 ± 0.006; p = 0.0007) (Fig. 4B). On the other hand, no significant changes in SPP levels were observed. Our laboratory has previously demonstrated that nuclear HO-1 localization increases after hemin treatment. In accordance with the up-regulation of Capn and CtsB, we now observed the presence of a cleaved HO-1 band (28 kDa) after hemin treatment of LM3 cells (control: 0.527 ± 0.73 vs hemin: 1.049 ± 0.7; p = 0.047) that is not present in vehicle-treated cells (Fig. 4C).

### Hemin treatment modulates focal adhesion- and epithelial-mesenchymal transition-related proteins

**Figure 5:**
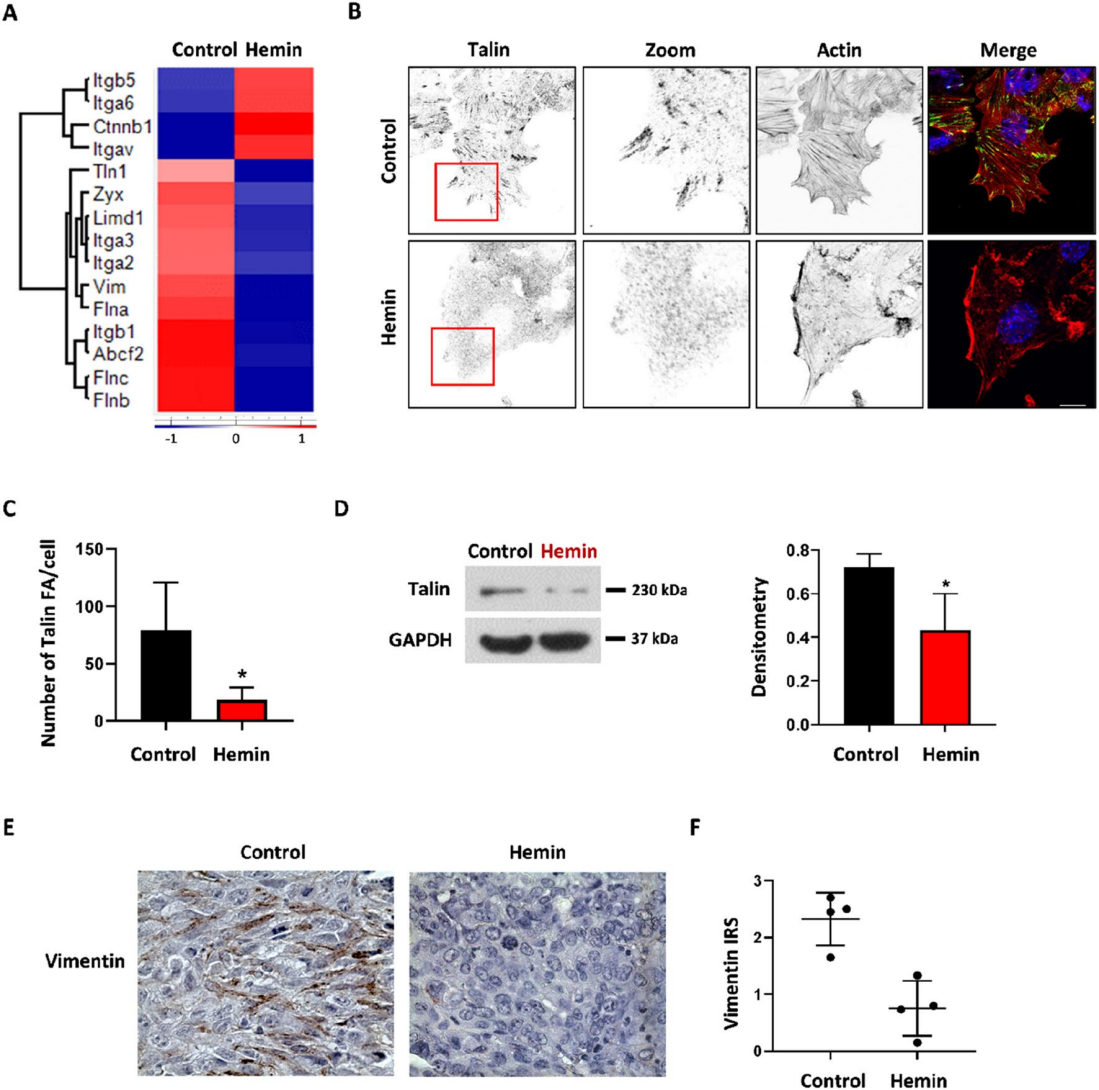
Hemin treatment affects adhesion- and EMT-related proteins in breast cancer cells. (**A**) Hierarchical cluster analysis shows adhesion- and EMT-protein expression after hemin treatment compared to control cells. Z-scores of median MS intensities are shown (n = 7). (**B**) Confocal microscopy images of Talin1 and F-actin distribution in vehicle- and hemin-treated LM3 cells are shown in grey scale. The merged images show an overlay of Talin (green), Actin (red) and DAPI (blue) staining. Scale bars 10 µm. (**C**) The bar graph shows the number of focal adhesions (FA) staining with Talin per cell after vehicle- or hemin-treatment (*p < 0.05; Student's T-test). (**D**) Western blot shows Talin protein expression after hemin treatment. GAPDH was used as loading control. Right: densitometric quantification of Talin/GAPDH ratio, (*p < 0.05; Student's T-test). (**E**) Immunohistochemistry of vimentin protein expression in LM3 syngeneic mouse model biopsies, comparing vehicle- and hemin-treated mice is shown. (**F**) Vimentin IRS quantification of vehicle- versus hemin-treatment (n = 4) (*p < 0.05; Student's T-test).

We have previously demonstrated that hemin treatment impairs migration and invasion processes by modulating pathways involved in epithelial-mesenchymal transition (EMT)^16^. Adhesion- and cytoskeleton-related proteins are involved in EMT and are also targets of various cancer treatments^26,27^. Since the EMT protein marker vimentin and several adhesion proteins were identified in the bioinformatic analyses as modulated by hemin treatment, we sought to corroborate these results by western-blot and immunofluorescence studies. The hierarchical cluster analysis showed that hemin induces an over-expression of αv, α6 and β5 (Itgav, Itga6 and Itgb5) integrin and under-expression of integrins β1, α2 and α3 (Itgb1, Itga2 and Itga3) (Fig. 5A). Talins (Tln), which are focal adhesion proteins that bind and activate integrins, were downregulated after hemin treatment. Similarly, other proteins that are also part of focal adhesions and important for cell migration, such as proteins with LIM domain (Limd1 and Zyx), and the filamin actin-binding proteins family (Fln a, b, c) were also downregulated following hemin treatment (Fig. 5A)^28,29^. In order to further study focal adhesion, immunofluorescent staining of talin-1 was performed. The immunofluorescence studies demonstrate that talin-1 decreases with hemin treatment (control: 79.33 ± 36 vs hemin: 18.66 ± 9.3; p = 0.046) (Fig. 5B,C). In addition, z-stacks images and orthogonal view from different planes (x/y, x/z or y/z) of talin immunostaining are shown in supplementary Fig. S3B. Furthermore, we confirmed the downregulation of talin expression after hemin treatment by Western blot (control: 0.72 ± 0.05 vs hemin: 0.434 ± 0.15; p = 0.0426) (Fig. 5D).

We had previously described that hemin increases E-cadherin levels and β-catenin membrane association, as well as a decrease in vimentin protein expression^16^. Vimentin is a major constituent of the intermediate filaments in mesenchymal cells and is usually overexpressed in BC cells, especially in those that have metastatic behaviour^30^. In agreement with these results, we observed in our hierarchical cluster analysis a decrease in vimentin (Vim) and an increase in β-catenin (Ctnnb1) protein levels after LM3 cells hemin treatment (Fig. 5A). Therefore, by performing vimentin IHC in tumour tissues from the animal model we corroborated the MS results. Indeed, a decrease in vimentin staining in tumours obtained from hemin-treated animals was observed (control: 2.325 vs hemin: 0.753 IRS; p < 0.05) (Fig. 5E,F).

These results show that modulation of the expression of EMT- and adhesion-related proteins by hemin could have an impact in the migration and metastatic behaviour of breast cancer cells.

### Hemin treatment modulates cytoskeletal-related proteins

**Figure 6:**
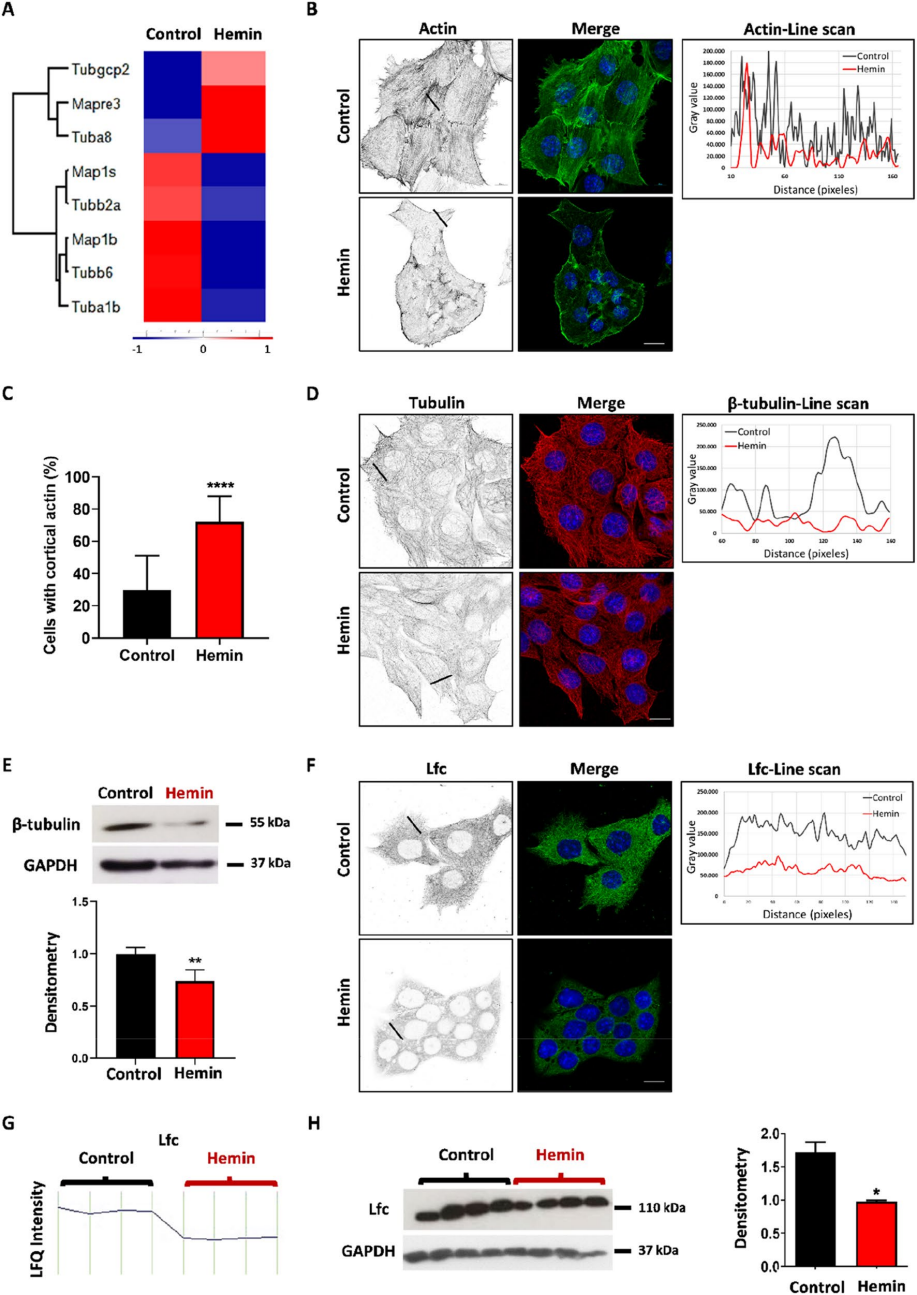
Hemin treatment affects cytoskeleton-related proteins in breast cancer cells. (**A**) Hierarchical cluster analysis shows the cytoskeleton-related proteins that were significantly modulated after hemin treatment, as detected by MS. The Z-scores of median MS intensities (Control, n = 7; Hemin, n = 7) are colour coded to show relative protein abundance. (**B**) Superresolution images of actin filaments (green) and nuclei (DAPI, blue) in LM3 cells. The right graph compares the line scan analysis of actin filament fluorescent intensity in vehicle- (black line) versus hemin- (red line) treated cells. (**C**) Bar graph shows the cortical actin cell quantification (****p < 0.0001; Student's T-test) (n > 400 cells per condition). (**D**) Superresolution images of β-tubulin (red) and nuclei (DAPI, blue) in LM3 cells. The right graph compares the line scan analysis of β-tubulin fluorescent intensity in vehicle- (black line) versus hemin-(red line) treated cells. (**E**) Western blot shows β-tubulin expression in LM3 cell lysates. GAPDH was used as loading control. Bottom: densitometric ratio of β-tubulin and GAPDH (**p < 0.01; Student's T-test), (n = 3 independent experiments). (**F**) Superresolution images of Lfc (green) and nuclei (DAPI, blue). Scale bars: 10 µm. Right: line scan of Lfc intensity and distribution, control versus hemin treated-cells. (**G**) LFQ intensity profile plot of Lfc (Arhgef2) protein expression in LM3 cells treated or not with hemin. (H) Western blot of LM3 cell lysates. Right: densitometry ratio of Lfc and GAPDH (*p < 0.05; Student's T-test), (n = 3 independent experiments).

Microtubules and actin filaments are essential for many biological processes, such as cell division, polarity, signalling and migration^31,32^. Furthermore, the cytoskeleton proteins have been a frequent target of anti-cancer drugs^33^. Therefore, we studied if hemin treatment had an effect in cytoskeletal-related proteins. We observed in the hierarchical cluster analysis a decrease in tubulin-associated proteins (Tubb2a, Tubb6, Tuba1b, Map1s, Map1b, Arhgef2) in LM3 cells after hemin treatment (Fig. 6A). In addition, orthogonal projection and confocal z-stacks images of actin immunostaining showed significant changes in LM3 stress fibers distribution following hemin treatment (Supplementary Fig. S3A). While control cells had more ventral and dorsal actin filaments, hemin treatment induced a loss of ventral actin filaments and an increase in cortical actin (control: 29.8 ± 27 vs hemin: 72 ± 9.3; p < 0.0001) (Fig. 6B,C). Since adequate dynamics and cell distribution of actin filaments are essential for cell migration, these results are in agreement with the impairment observed in breast cancer cell migration after hemin treatment^16^. Furthermore, by means of immunofluorescent staining, we observed a reduction in microtubule formation (Fig. 6D and supplementary Fig. S3C), and by means of western-blot a decrease in total β-tubulin protein expression after LM3 hemin treatment (control: 0.997 ± 0.07 vs hemin: 0.741 ± 0.09; p < 0.01) (Fig. 6E).

Cytoskeleton reorganisation and dynamics are tightly regulated by Rho-GTPases family^32^. Guanine nucleotide exchange factors (GEFs) promote Rho-GTPases activation by exchanging GDP for GTP^34^. We observed from MS data analysis that a particular GEFs, Arhgef2 (Lfc in mice, ortholog of GEF-H1 in humans), was downregulated after hemin treatment in LM3 cells (Fig. 6A). Lfc was first described as an oncogene and it was shown to be the link between RhoA-GTPases, microtubule and actin polymerization processes^35,36^. Lfc and p190RhoGEF have been found to localise with microtubules and stabilise these filaments, although only Lfc activity is microtubule-polymerization-dependent^37,38^. We observed a decrease in Lfc microtubule localization after LM3 hemin treatment (Fig. 6F and supplementary Fig. S3D) and LFQ intensity levels (Fig. 6G). We confirmed this result by western blot protein expression (control: 1.717 ± 0.16 vs hemin: 0.977 ± 0.03; p = 0.017) (Fig. 6H). Consequently, hemin treatment could be acting through Lfc Rho-GEF expression and its subcellular localization to modulate RhoA-GTPases activation and cytoskeleton polymerization.

### Hemin treatment changes the expression profile of iron metabolism-related proteins

**Figure 7:**
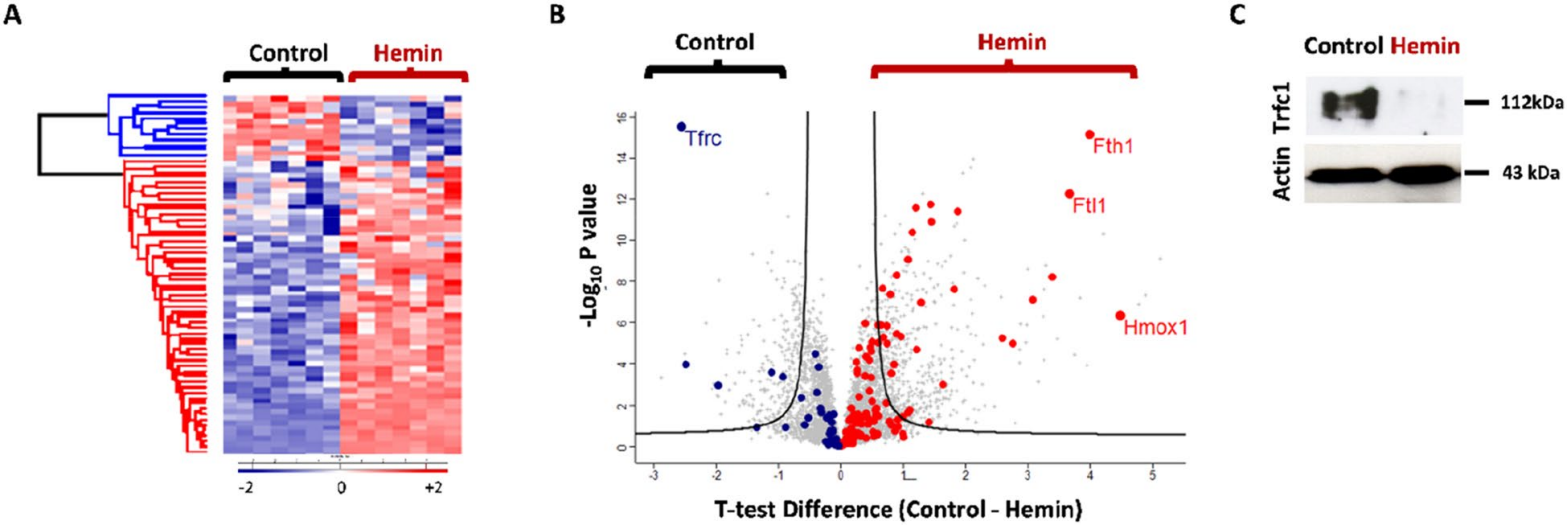
Effects of hemin on iron-related proteins at the proteome level. (**A**) Hierarchical cluster analysis of Student's T-test-significant iron-related proteins (GOMF and GOBP). Red cluster shows that 55 out of 67 iron-related proteins were upregulated after hemin treatment. (**B**) Volcano plot comparing protein expression in vehicle- and hemin-treated samples. Iron-related proteins are shown as dots and total proteins as grey crosses. The proteins that were up-regulated upon hemin treatment are shown as red dots and those that were down-regulated are shown as blue dots. Most significant proteins were text labels as ferritin heavy chain 1 (Fht1), ferritin light chain (Ftl1), HO-1 (Hmox1) and transferrin receptor (Tfrc). The curve is derived at FDR = 0.05 and S0 = 0.5 and T-Test was applied. (**C**) Western blots show the Tfrc protein expression. Actin was used as loading control.

We have previously demonstrated that hemin activation of LM3 BC cells results in an induction of cytoplasmic iron^16^. Since alterations of iron metabolism have been related to the pathophysiological mechanisms of BC, in this work we analysed if hemin treatment produces a regulation of proteins that mobilise iron^39^. The hierarchical cluster heatmap shows an increase in total hemin-upregulated iron related proteins compared with vehicle treatment (Fig. 7A). Volcano plot showed that ferritin (Fth1, Ftl1), a protein which stores iron inside the cell, is overexpressed after hemin treatment compared with control cells (Fig. 7B). In addition, the levels of transferrin receptor 1 (Tfrc1) which transports iron inside the cell decreased following hemin treatment (Fig. 7B). The latter was also corroborated by western blot (Fig. 7C). These results show that hemin modulates iron storage and iron transport proteins in BC cells.

### Hemin treatment alters the expression profile of proteins related to lipid metabolism and generates neutral lipid accumulation

**Figure 8:**
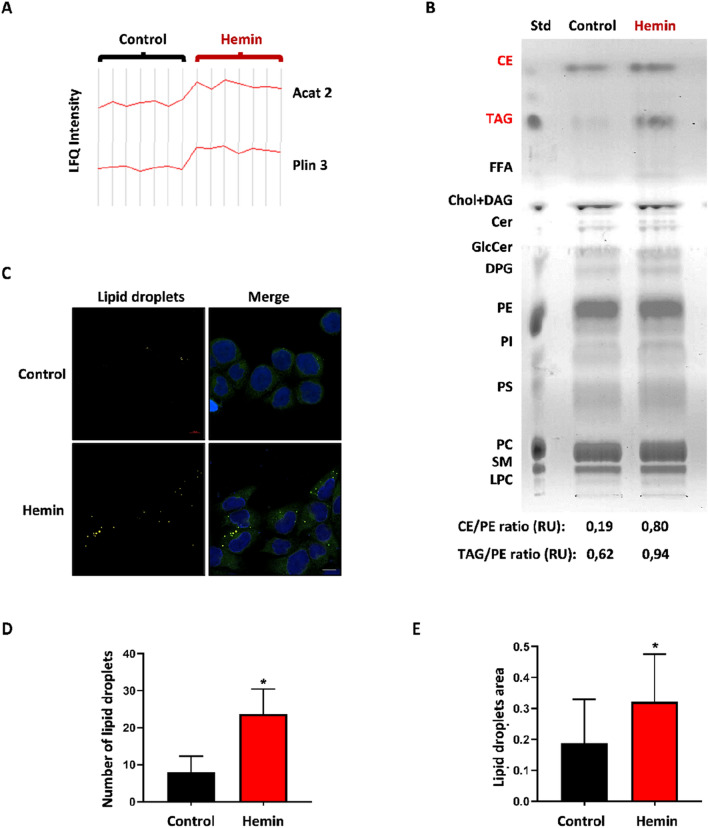
Hemin increased lipid metabolism-related proteins (**A**) LFQ intensity profile plot of acetyl-CoA acetyltransferase (Acat2) and periplin-3 (Plin3) enzymes detected by MS, in vehicle- vs hemin-treated cells. (**B**) Thin layer chromatography to resolve the following lipids after the treatments: cholesterol esters (CE), triglycerides (TAG), phosphatidylethanolamine (PE), phosphatidylinositol (PI), phosphatidylserine (PS) and sphingomyelin (SM). Shown is the lipid standard (STD). Bottom: Relative density values ratio of CE and TAG levels using PE as a loading control, expressed in relative units (RU). (**C**) Confocal images of nile red staining of lipid droplets (yellow) and nuclei (DAPI, blue) in LM3 cells treated with hemin or vehicle. Scale bars: 10 µm. (**D**) Quantification of lipid droplet number per field (*p < 0.05; Student's T-test). (**E**) Quantification of lipid droplet size using Image J software (*p < 0.05; Student's T-test).

Lipid metabolic enzymes are frequently altered in breast cancer cells^40^. Interestingly, the LFQ intensity profile plot showed an up-regulation of lipid metabolism-related proteins, specifically an increase in acetyl-CoA acetyltransferase (Acat2) and periplin-3 (Plin3) (Fig. 8A). Given the increased expression levels of lipid metabolism-related proteins, we evaluated if these observations had an effect on the lipid profile of LM3 cells exposed to hemin. Remarkably, we found a neutral lipid build-up, mainly due to a rise in triacylglycerols (TAG) and cholesterol esters (CE) levels (Fig. 8B). Normally, these lipids are stored on cytosolic lipid droplets, these being functional organelles involved in lipid metabolism and homeostasis. Thus, using the specific nile-red staining, we studied if this increment of neutral lipids was associated with an increase in the number of lipid droplets in hemin-treated LM3 cells (Fig. 8C). Interestingly, as expected, the number (control: 8 ± 4 vs hemin: 23.66 ± 6.5; p = 0.028) and size (control: 0.189 ± 0.13 vs hemin: 0.322 ± 0.15; p = 0.025) of lipids droplets increased after hemin treatment as compared with vehicle-treated cells (Fig. 8D,E).

## Discussion

In this work we have studied by mass spectrometry (LC–MS/MS) the effect of hemin treatment on the proteome of the LM3 mammary carcinoma cell line. By using bioinformatic tools we further analysed the molecules and pathways that were modulated. In addition, we corroborated, by means of biochemical and cellular studies, the most interesting proteins/pathways related to mammary cancer. Our study has shown that this heme-protoporphyrin modulates the expression of 2230 proteins, which are mostly related to signal transduction, cytoskeletal dynamics, cell migration, and iron- and lipid-metabolism.

As expected, HO-1 was one of the proteins strongly induced by hemin. We have previously demonstrated that this enzyme has an important antitumoral effect in mammary carcinoma^16^. HO-1 has been first characterised as a protein associated with the endoplasmic reticulum (ER) but was later found localised in other subcellular compartments including the cell nucleus where it can function as a co-transcriptional factor, independent of its enzymatic activity^9^. Indeed, we have demonstrated that nuclear HO-1 lacks enzymatic activity^16^. It has been demonstrated that the migration of HO-1 to the nucleus requires the cleavage of the C-terminal end, which allows it to be released from the ER-membrane in which it is anchored. In relation to this, we have herein observed by MS that two of the three previously reported proteases known to cut the C-terminal end of HO-1, namely cathepsin and calpain, are overexpressed after pharmacological modulation of BC cells with hemin. This overexpression is accompanied by an increase in the HO-1 cleaved band. However, further experiments are needed to conclude that the increase in proteases expression by hemin is the cause of the increase in the HO-1 cleaved band. We have recently proposed that the known dual role of HO-1 in cancer is dependent on the cytoplasmic or nuclear localization^21^. Hence, hemin could be regulating the HO-1 subcellular localization, and therefore, through cathepsin and calpain induction, its function.

In this work, we have also demonstrated that hemin increases the levels of PTEN, a tumour suppressor gene and a negative regulator of PI3K, suggesting a down regulation of the pro-survival and pro-proliferative PI3K/Akt signalling pathway. The role of PTEN is very well known in breast cancer. Indeed, its loss/mutation was reported in different breast cancer subtypes and was associated with worse clinical outcome (Cancer Genome Atlas Network).

In addition, we had previously demonstrated that hemin down regulates NF-kB pathway, a fact which may account for the impairment of the EMT, angiogenesis and apoptosis processes in breast cancer^16^. It is well known the crosstalk between PI3K/Akt and NF-kB signalling by which Akt inhibits this transcription factor, thus impairing its anti-apoptotic effect. Therefore, if PTEN is augmented by hemin, Akt is downregulated and cannot inhibit NF-kB, thus leading to an increase in the apoptotic rate. We therefore suggest that this PTEN/PI3K/Akt/NF-kB axis may be implicated in hemin antitumor effect in breast cancer.

We also found that hemin induces an up regulation of Smad2/3, a downstream effector in TGF-β signalling pathways. The role of TGF-β in tumorigenesis depends on tumour stage^41^. Since TGF-β is a potent cell division inhibitor, abnormalities in TGF-β signalling result in carcinogenesis. A tumour suppressor role for Smad2 has been proposed in breast cancer metastasis. Indeed, a reduced phosphorylated Smad2 staining was associated with a reduced overall survival in patients with stage II breast cancer^42^. Our group had previously demonstrated that wild type p53 is required for HO-1 antitumor effects induced by hemin in colorectal cancer^15^. Bearing this in mind, it is striking that the tumour suppressor protein p53 was down regulated after hemin treatment. As TP53 gene is frequently mutated in human breast cancer (Cancer Genome Atlas Network) it will be necessary to study the mutational status of this protein in LM3 cell line to be able to explain this result.

In order to migrate and invade through the extracellular matrix, processes which are necessary for the metastatic process, cancer cells make use of their cytoskeleton^43^. Interestingly, within the group of proteins that were strongly modulated, those which are linked to the cytoskeleton stood out. After treatment with hemin, the proteins involved in focal adhesion formation were found to be decreased, as well as the actin and tubulin filaments and their stabilising proteins. This indicates that hemin alters the mechanism responsible for allowing cell migration, which may account for the decrease in the migratory capacity of hemin-treated LM3 cells. As Ridley et al. have previously described, the family of Rho-GTPases are the main proteins responsible for the regulation of the cytoskeleton^44^. Importantly, in this work we also demonstrated by hierarchical cluster analysis that the Lfc oncogene protein, which is a RhoA-GTPase activator, decreases after hemin treatment. This would lead to a decrease in the activation of RhoA-GTPases responsible for regulating the dynamics of the actin and tubulin cytoskeleton^26,36^. In this way, the turnover of focal adhesions would be affected, thus accounting for the impairment in cell migration^45^. Importantly, the MS results were further corroborated by western-blot, immunofluorescence and microscopy, showing a decrease in RhoA activator (Lfc), focal adhesion- and cytoskeleton-related proteins following hemin treatment.

Of the 1309 overexpressed proteins after hemin treatment, we observed two upregulated protein clusters, one of them related to lipid- and the other one to iron-metabolism. As far as we know, this study shows for the first time that hemin can be linked to lipid metabolism. Lipid metabolism is frequently altered in cancer cells^40^. Specifically for this work we have observed an increase in the number and size of lipid droplets that correlates with an increase in CE and TAG. These results are in agreement with the increased expression of the Plin3 protein, a protein which plays a crucial role in the stabilisation and formation of intracellular lipid droplets^46^. In turn, the increased CE observed could be related to the increased expression of Acat2 also observed by MS, since this enzyme catalyses the acylation of free cholesterol. Taken together, these results may partly explain the antiproliferative effect of hemin. Breast cancer cells have an active lipid metabolism, in particular, de novo lipogenesis that provides the substrates (i.e., fatty acids and acyl-CoAs) to biosynthesize the different classes of membrane lipids required to maintain the high rate of cell division and energetic needs of tumours in progression^40^. Thus, in cells exposed to hemin, the accumulation of neutral lipids could reflect their lack of utilisation in their usual metabolic pathways (i.e., membrane phospholipid biosynthesis, fatty acid oxidation).

In relation to iron metabolism, our results have shown that hemin modulates iron storage and iron transport proteins in LM3 BC cells. It is well known that hemin activates HO-1 which produces the breakdown of heme, thus increasing iron levels. Accordingly, we observed an increase in ferritin, which stores iron inside the cell. Consequently, the transferrin receptor, which is responsible for introducing iron inside the cells, is downregulated. High levels of intracellular iron induce ROS formation, which, in turn, induces apoptosis^47^. This induction in cell death might be partly responsible for the antitumor effects observed for hemin in BC^16^. Furthermore, iron-dependent accumulation of oxidatively damaged phospholipids (i.e., lipid peroxides) has been shown to induce ferroptosis^48^. Importantly, it has been demonstrated that hemin displays a dual function in enhancing the radiosensitivity of ferroptosis in lung cancer cells while promoting cell survival in normal lung cells^49^. In breast cancer cells, it has been reported that pharmacological induction of HO-1 causes cell death by ferroptosis^50^. In this regard, a recent study of our group demonstrated that HO-1 modulation increases cellular iron levels through deregulation of the iron proteins which generates greater lipid peroxidation that is closely linked to ferroptosis^51^.

**Figure 9:**
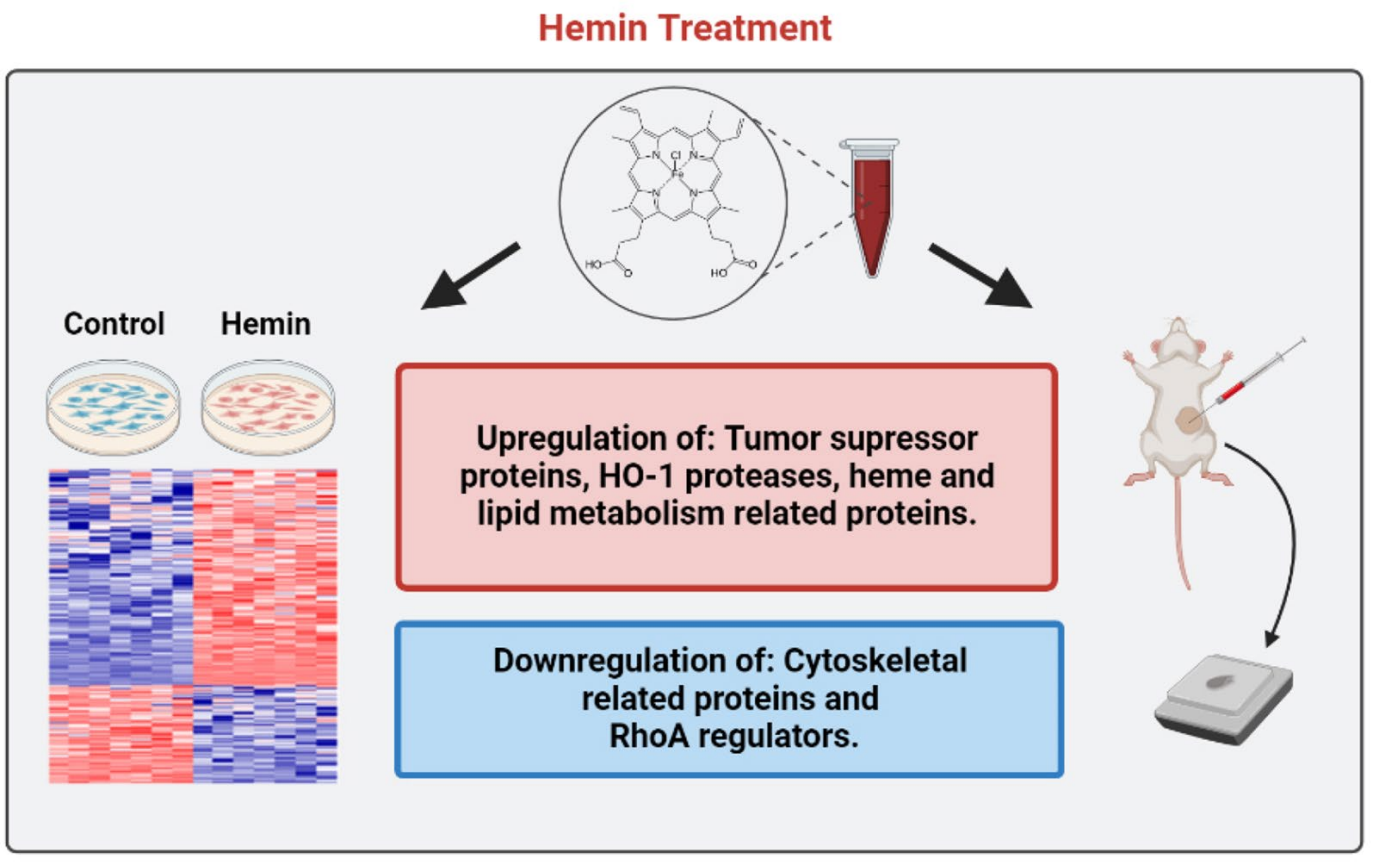
Graphical abstract including the main findings of the work.

We have previously shown that hemin impairs tumour progression in mammary cancer, mostly through the action of HO-1^16^. The present work in which we have analysed the hemin effect on the proteome of a breast cancer cell line has partly confirmed the antitumoral hemin effect, and has also shed light on the various molecular mechanisms through which hemin exerts these antitumoral effects (Fig. 9).

## Materials and methods

### Cell culture

LM3 is a hormone-independent tumour cell line derived from a murine mammary adenocarcinoma that spontaneously arose in BALB/c mice^52^. This cell line is a generous gift from Instituto de Oncología Dr. Ángel H. Roffo, Buenos Aires, Argentina. The cells were cultured in Minimum Essential Medium (MEM, INVITROGEN) supplemented with 5% fetal bovine serum (NATOCOR) and 1% antibiotic–antimycotic (GIBCO).

### Mass spectrometry (MS)

LM3 cells were treated with hemin (80 µM) and its respective control (vehicle: 0.4 mL of NaOH 0.5N and 0.5 mL Tris–HCl 0.5N) for 24 h. This concentration was chosen based on our previous results in BC^16^. Cells were lysed with radio-immunoprecipitation assay (RIPA) lysis buffer (25 mM Tris–HCl, pH 7.5; 150 mM NaCl; 1% TX100; 0.2% SDS; 0.5% sodium deoxycholic acid; protease and phosphatases inhibitors) on ice, following Bradford (BioRad) protein quantification. The same amount of protein was incubated with Laemmli buffer for five minutes at 95 °C. Proteins were separated by SDS–polyacrylamide gel electrophoresis on a 4–15% gradient gel. The gel was stained with Coomassie using the GelCode Blue Safe Protein Stain reagent (Thermo) and used for mass analysis (Supplementary Fig. S1A,B). Two independent experiments were performed (seven biological replicates were processed for control and seven for hemin condition). The electrophoresis-stained gels were sent to the Mass Spectrometer core facility at Max Planck Institute of Biochemistry in Germany.

Sample preparation—Gel lanes of interest were excised, chopped and washed twice with 150 µL of de-staining buffer (25 mM ammonium bicarbonate, 50% ethanol). Gel pieces were dehydrated twice in 150 µL of 100% ethanol and the gel pieces were dried by vacuum centrifugation. Then, 50 µL of digestion buffer (25 mM Tris HCl, 10% acetonitrile, 10 ng/µL of trypsin) was added. After incubation for 20 min on ice, 50 µL of ammonium bicarbonate buffer (25 mM) was added and the gel pieces were incubated at 37 °C overnight.

Peptides in the supernatant were collected and more peptides were extracted from the gel pieces by repeated incubation of the gel pieces at 25 °C in 100 µL of extraction buffer (3% TFA, 30% acetonitrile), with a subsequent centrifugation and collection of the supernatants. Finally, the gel pieces were dehydrated by incubation at 25 °C in 100 µL of 100% acetonitrile and the supernatant was unified with the supernatants from previous extraction steps. Acetonitrile was removed by vacuum-centrifugation and 70 µL of 2 M Tris HCl as well as 10 mM tris (2-carboxyethyl) phosphine and 40 mM 2-chloroacetamide was added. After incubation for 30 min at 37 °C, peptides were acidified to 1% TFA and desalted using Stage Tips.

Liquid Chromatography with tandem mass spectrometry (LC–MS/MS) data acquisition—Purified and desalted peptides were loaded onto a 30 cm column (inner diameter: 75 microns; packed in-house with ReproSil-Pur C18-AQ 1.9-micron beads, Dr. Maisch GmbH) via the autosampler of the Thermo Easy-nLC 1000 (Thermo Fisher Scientific) at 60 °C. Using the nanoelectrospray interface, eluting peptides were directly sprayed onto the benchtop Orbitrap mass spectrometer Q Exactive HF (Thermo Fisher Scientific).

Peptides were loaded in buffer A (0.1% formic acid) at 250 nl/min and percentage of buffer B (80% acetonitrile, 0.1% formic acid) increased from 2 to 30% over 120 min, followed by an increase to 60% over 10 min and then 95% over the next 5 min. Percentage of buffer B was maintained at 95% for another 5 min.

The mass spectrometer was operated in a data-dependent mode with survey scans from 300 to 1650 m/z (resolution of 60,000 at m/z = 200), and up to 10 of the top precursors were selected and fragmented using higher energy collisional dissociation (HCD with a normalised collision energy of value of 28). The MS2 spectra were recorded at a resolution of 15,000 (at m/z = 200). AGC target for MS and MS2 scans were set to 3E6 and 1E5 respectively within a maximum injection time of 100 and 60 min for MS and MS2 scans respectively. Dynamic exclusion was set to 30 m.

Data Analysis—Raw data were processed using the MaxQuant computational platform (version 1.6.5.0) (https://www.nature.com/articles/nbt.1511) with standard settings applied. Shortly, the peak list was searched against the Uniprot database of Mus musculus (55.466 entries) with an allowed precursor mass deviation of 4.5 ppm and an allowed fragment mass deviation of 20 ppm. MaxQuant, by default, enables individual peptide mass tolerances, which was used in the search. Cysteine carbamidomethylation, methionine oxidation and N-terminal acetylation were set as variable modifications. Proteins were quantified across samples using the label-free quantification algorithm in MaxQuant as label-free quantification (LFQ) intensities.

### Bioinformatics analysis

Bioinformatic analysis and heatmaps generation were made with the free access program Perseus, developed at Max Planck Biochemistry institute by Dr. Cox (https://maxquant.net/perseus/)^53^. The data corresponding to the biological replicates were filtered, based on valid values. Multiple testing corrections were performed. Those proteins that were significantly different (*p < 0.05) between the hemin-treated and vehicle-treated cells were selected after applying the Student's T-test. The free access program STRING was used to generate the associated protein networks (https://string-db.org/). The gene ontology (GO) analysis of significant proteins was performed using DAVID webserver (https://david.ncifcrf.gov/). The resulting categories of biological processes, molecular functions and Kyoto Encyclopaedia of Genes and Genomes (KEGG) pathways dataset were further sub-clustered into eleven global categories^54^.

### Western blot

LM3 cells were seeded and grown sub-confluent until treatment with 80 µM hemin or vehicle. Immunoblotting was performed as previously described^23^, incubating membranes with anti-HO-1 (1:1000; Enzo), anti-GEF-H1 (1:2000; Abcam), anti-actin (1:1000; Santa Cruz Biotechnology, Inc.), anti-tubulin (1:1000; Santa Cruz Biotechnology, Inc.), anti-PTEN (1:1000; Santa Cruz Biotechnology, Inc.), anti-Smad2/3 (1:1000; Santa Cruz Biotechnology, Inc.), anti-p53 (1:500; Santa Cruz Biotechnology, Inc.), anti-vimentin (1:1000; Santa Cruz Biotechnology, Inc.), anti-GAPDH (1:5000; MERK) and anti-transferrin receptor 1 (1:500, Santa Cruz Biotechnology, Inc.). To differentiate the HO-1 cleaved band from the native HO-1, we performed a 10% gel and ran it until the running front fell off. For talin we performed an 8% gel. For the rest of the proteins we performed a 12% gel. Western blot bands relative density was calculated with Image J free software and expressed in relative units (RU) (https://imagej.nih.gov/ij/). Uncropped western blots are shown in supplementary Fig. S4.

### Immunofluorescence

LM3 cells were seeded and grown 80% confluent until treatment with 80 µM hemin or vehicle. Immunofluorescence was performed as previously described^23^. Rabbit anti-talin (1:100, Sigma), anti-tubulin-Cy3 (1:300, Sigma) and anti-GEF-H1 (1:500, Abcam) primary antibodies were used. For actin filaments, we used staining with Phalloidin-TRITC (1:300; Invitrogen) for 1 h at room temperature. Secondary Alexa 546 and 488 fluoro-conjugated antibodies (Molecular Probes) were used. DAPI was used to stain the nucleus. The coverslips were mounted on slides with mowiol mounting medium and we obtained superresolution and confocal z-stacks images in Zeiss LSM900 microscope with an Airyscan 2 module. Orthogonal x/y, x/z or y/z projection views were performed with Zeiss ZEN Software. Image intensity and protein distribution were analysed with Image J free software.

### Lipid extraction and thin layer chromatography

Lipid extracts were prepared following the method of Bligh and Dyer. Aliquots from these extracts were taken to determine the total phospholipid (PL) content in the samples by measuring the amount of lipid phosphorus. In order to study the polar and neutral lipid profile of the LM3 cells in vehicle- and hemin-treated cells, a single mono-dimensional high-performance thin layer chromatography (HPTLC) was performed. The same amount of lipid phosphorus (10 µg) per condition was taken from lipid extracts and spotted onto an HPTLC silica gel plate (Merck). The lipids were then resolved using a combination of solvents as follows: first, chloroform/methanol/acetic acid/water (50:37.5:3.5:2, v/v/v/v). The HPTLC was run up to approximately the middle of the plates to resolve the PL classes: lysophosphatidylcholine (LPC), sphingomyelin (SM), phosphatidylcholine (PC), phosphatidylinositol (PI), phosphatidylserine (PS), phosphatidylethanolamine (PE) and diphosphoglycerate (DPG). Then, after drying the plates, diethyl ether was applied, allowing it to overpass the solvent front by 1–2 cm in order to concentrate the neutral lipids into a single compacted band. Finally, hexane/ether (80:20, v/v) was run up to the top of the plates to resolve the neutral lipids: cerine, cholesterol + diacilglicerol (DAG), free fatty acids (FFA), triacylglycerols (TAG) and cholesterol esters (CE). A spot containing lipid standards was developed simultaneously to allow lipid identification. To visualise the bands the plate was exposed to iodine vapours. Bands relative density were calculated with Image J free software (analyse gel and plot profile), and expressed in Relative Units (RU).

### Lipid droplets staining

LM3 cells were seeded and grown 80% confluent until treatment with 80 µM hemin or vehicle. After treatments cells were washed with phosphate-buffered saline (PBS) and stained for 15 min with Nile Red (Molecular Probes), 1.5 μg/ml in serum free medium. Cells were stained with DAPI and then observed with LSM 900 (Zeiss) confocal microscope to visualise lipid droplets. Droplets number and size were analysed with Image J free software (analyse particles option).

### Animal studies

We used paraffin-embedded tumour tissues obtained from a subcutaneous implant model of LM3 cells in BALB/C syngeneic mice, tissues which had been previously obtained in our laboratory. The procedure is described in Gandini et al.^16^.

### Immunohistochemical staining

Immunohistochemical (IHC) staining was performed as previously described^23^. In brief, slides were deparaffinized in xylene and rehydrated in a series of decreasing ethanol dilutions and PBS. They were incubated in 3% hydrogen peroxide to quench endogenous peroxidase and were blocked with 2% bovine serum albumin in PBS (blocking solution). Sections were then incubated overnight at 4 °C with anti-vimentin antibody (1:250; Santa Cruz Biotechnology, Inc); anti-Smad2/3 antibody (1:250; Santa Cruz Biotechnology, Inc), anti-PTEN antibody (1:250; Santa Cruz Biotechnology, Inc), and HO-1 (1:50; Enzo). Incubation with diluted biotinylated secondary antibody and VECTASTAIN ABC Reagent (Vector Laboratories, Inc.) was then performed. For negative controls, the primary antibody was replaced by the same isotype immunoglobulin. For positive controls, IHC for Smad2/3, PTEN and vimentin were performed in different organs. Diaminobenzidine/H_2_O_2_ was used as a substrate for the immunoperoxidase reaction. They were counterstained with hematoxylin and mounted with Permount (Fisher Scientific) for analysis by bright-field microscopy.

### Histopathologic evaluation of staining intensities

Vimentin, Smad2/3 and PTEN expression levels were scored semi-quantitatively based on the proportion of stained cells and the staining intensity, using the immunoreactive score (IRS), as previously described^23^.

### Statistical analyses

The GraphPad Prism version 8.0.0 for Windows was used for processing and statistical analysis of all data. The IRS analysis between groups was done with the Mann–Whitney U test. The applied statistical methods are specified in the figure legends.

## Supplementary Information

Supplementary Information 1.

Supplementary Information 2.

Supplementary Information 3.

Supplementary Information 4.

Supplementary Information 5.

## Data Availability

The mass spectrometry proteomics data have been deposited to the ProteomeXchange Consortium via the PRIDE partner repository (http://www.ebi.ac. uk/pride/archive/) with the dataset identifier PXD036701.

## Abbreviations

BC: Breast cancer
HER2: Epidermal growth factor receptor
HO: Heme oxygenase
LC–MS/MS: Liquid chromatography-tandem mass spectrometry
LFQ: Label free quantification
FDR: False discovery rate
GO: Gene ontology
KEGG: Kyoto encyclopaedia of genes and genomes
HPTLC: High-performance thin layer chromatography
PL: Phospholipid
CE: Cholesterol esters
PE: Phosphatidylethanolamine
TAG: Triglycerides
PI: Phosphatidylinositol
PS: Phosphatidylserine
SM: Sphingomyelin
RU: Relative units
ROS: Reactive oxygen species
IHC: Immunohistochemical
IRS: Staining intensity score
PBS: Phosphate buffer saline
EMT: Epitelial-mesenchymal transition
ER: Endoplasmic reticulum
PTEN: Phosphatase and tensin homolog
IDH1: Isocitrate dehydrogenase 1
IDH2: Isocitrate dehydrogenase 2
RBL1: RB transcriptional corepressor like 1
SPP: Signal peptide peptidase
Capn: Calpain 1
CtsB: Cathepsin B
Tln: Talin
GEFs: Guanine nucleotide exchange factors
Rtf1: Transferrin receptor 1
Plin3: Periplin-3
Acat2: Acetyl-CoA acetyltransferase
