# Supplementary material for: Proteomic analysis of the effect of hemin in breast cancer
Source: Sci Rep. 2023 Jun 21;13:10091. doi: 10.1038/s41598-023-35125-4 (PMC10284804; doi:10.1038/s41598-023-35125-4)
Supplement: Supplementary file 1 — Supplementary Information 1. [file 41598_2023_35125_MOESM1_ESM.pdf]

Supplementary Figure 1

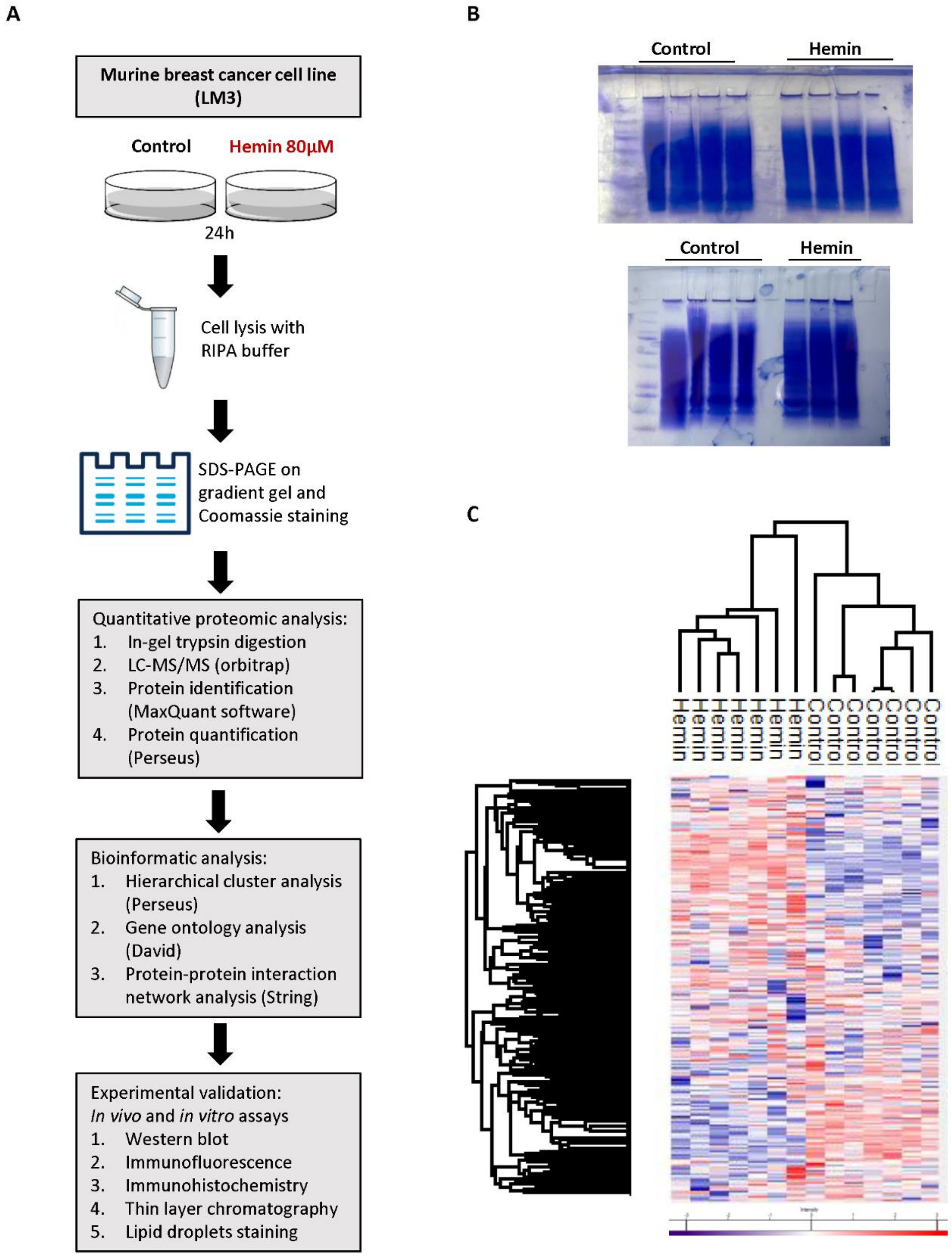

Supplementary Figure 1: (A) Experimental design for the proteome analysis of vehicle (control)- and hemin-treated LM3 cells. (B) Coomassie-stained polyacrylamide gradient gel of LM3 cells lysates. Gel lines were excised and digested for LC-MS/MS. Second line of bottom gel from control cells was not used in the analyses. (C) The resulting peptides were analysed by high-resolution MS and raw files processed using the MaxQuant software. Hierarchical clustering was generated with Perseus free software. Heatmap of MS intensities (n=7; 2 independent experiments) was colour coded to show relative protein abundance: shown in red are the up-regulated proteins, shown in blue are the down-regulated ones and shown in white are the proteins without change between the vehicle- and hemin-treated cells.
