# Supplementary material for: Proteomic analysis of the effect of hemin in breast cancer
Source: Sci Rep. 2023 Jun 21;13:10091. doi: 10.1038/s41598-023-35125-4 (PMC10284804; doi:10.1038/s41598-023-35125-4)
Supplement: Supplementary file 2 — Supplementary Information 2. [file 41598_2023_35125_MOESM2_ESM.pdf]

Supplementary Figure 2

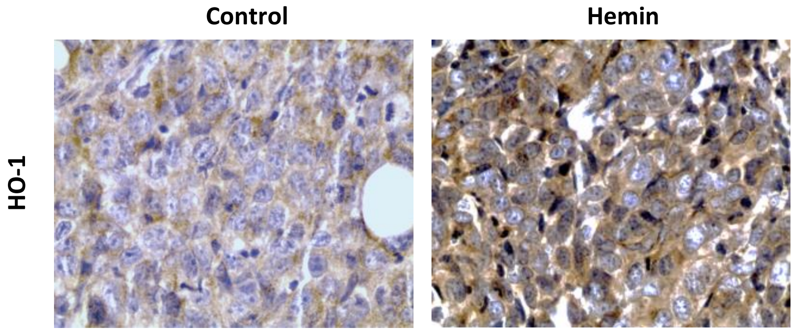

Supplementary Figure 2: Immunohistochemistry of HO-1 in LM3 biopsies of syngeneic mouse model treated with vehicle- and hemin- treated.
