# Supplementary material for: Proteomic analysis of the effect of hemin in breast cancer
Source: Sci Rep. 2023 Jun 21;13:10091. doi: 10.1038/s41598-023-35125-4 (PMC10284804; doi:10.1038/s41598-023-35125-4)
Supplement: Supplementary file 3 — Supplementary Information 3. [file 41598_2023_35125_MOESM3_ESM.pdf]

Supplementary Figure 3

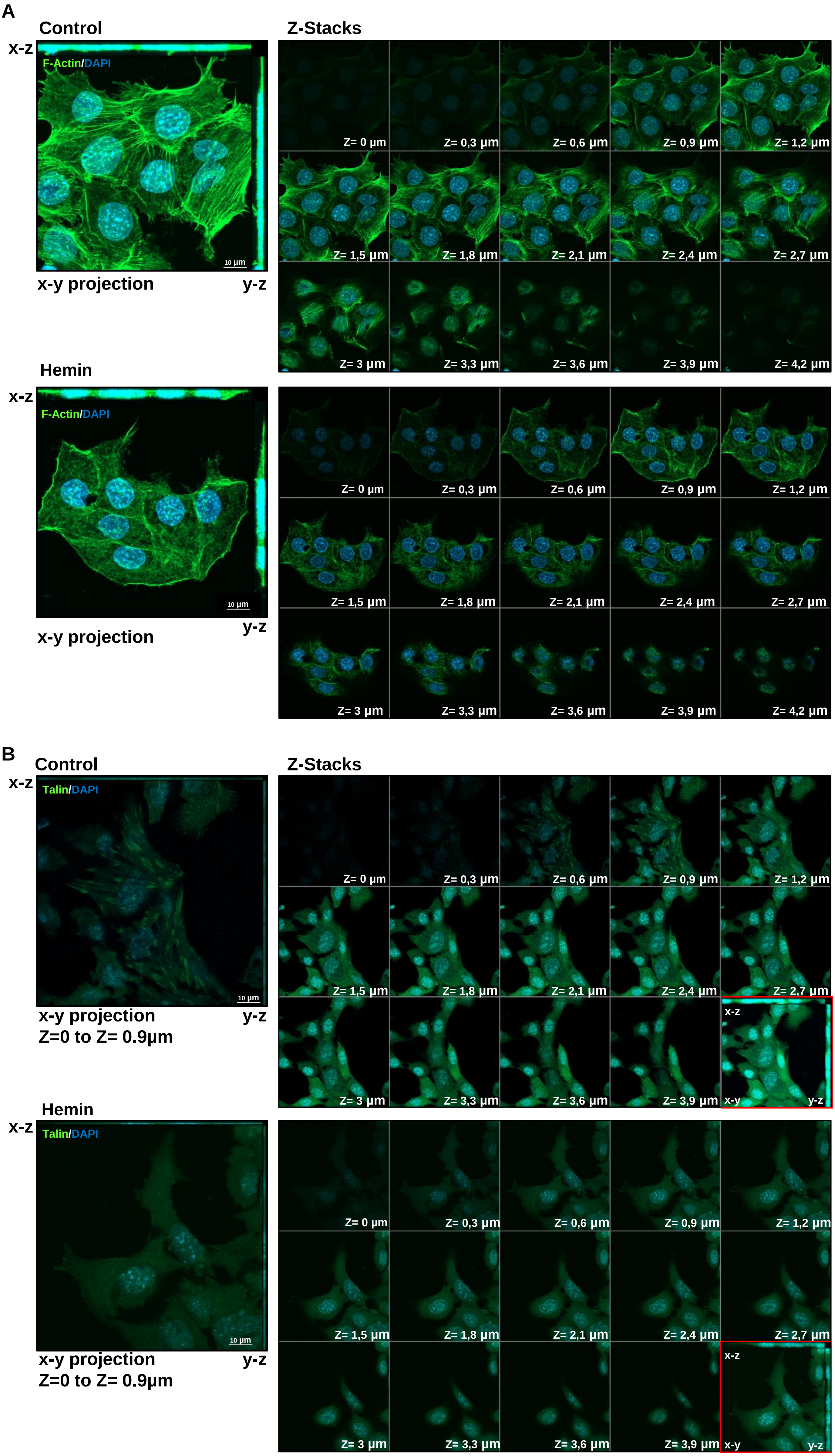

Supplementary Figure 3 continued

C

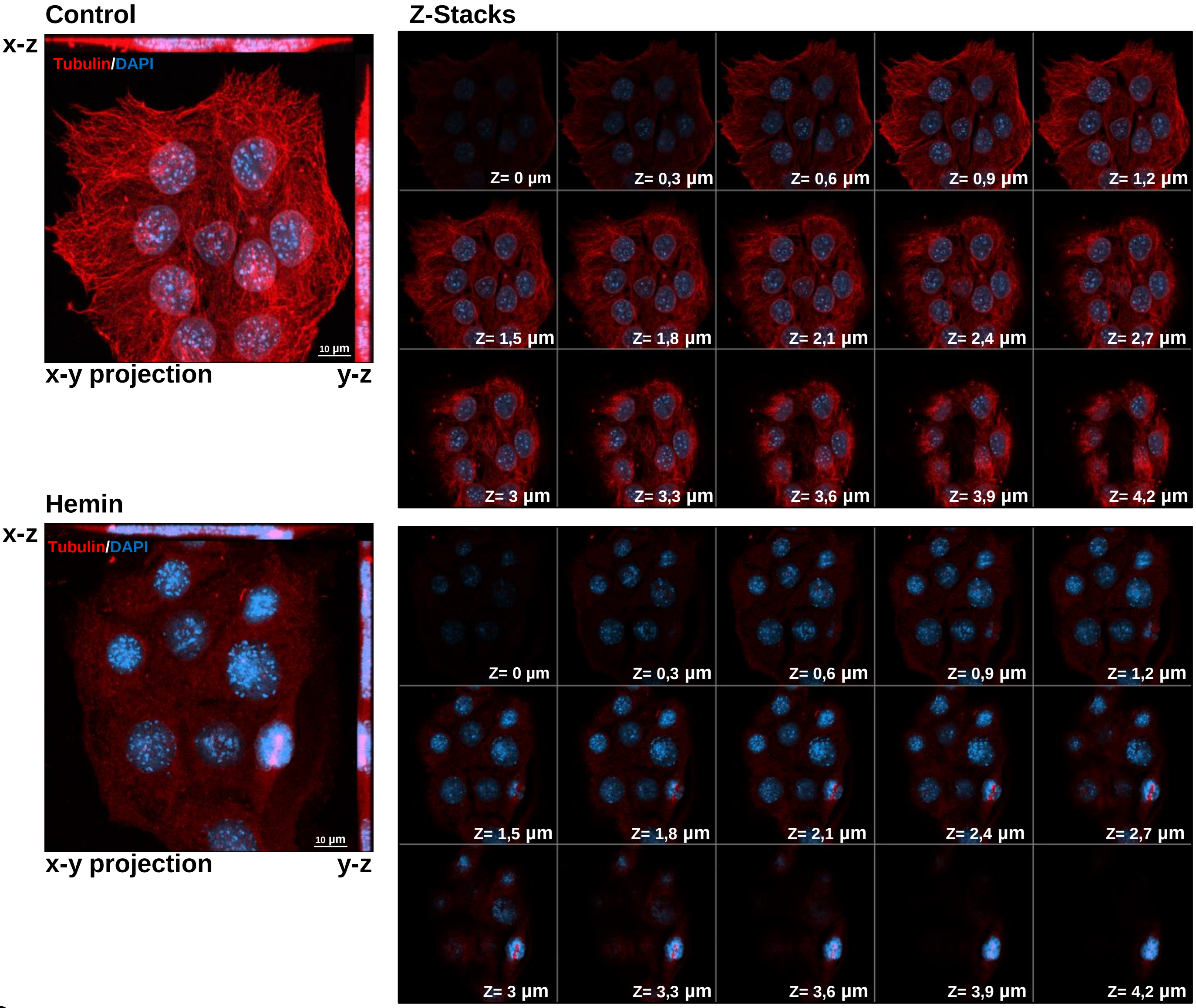

D

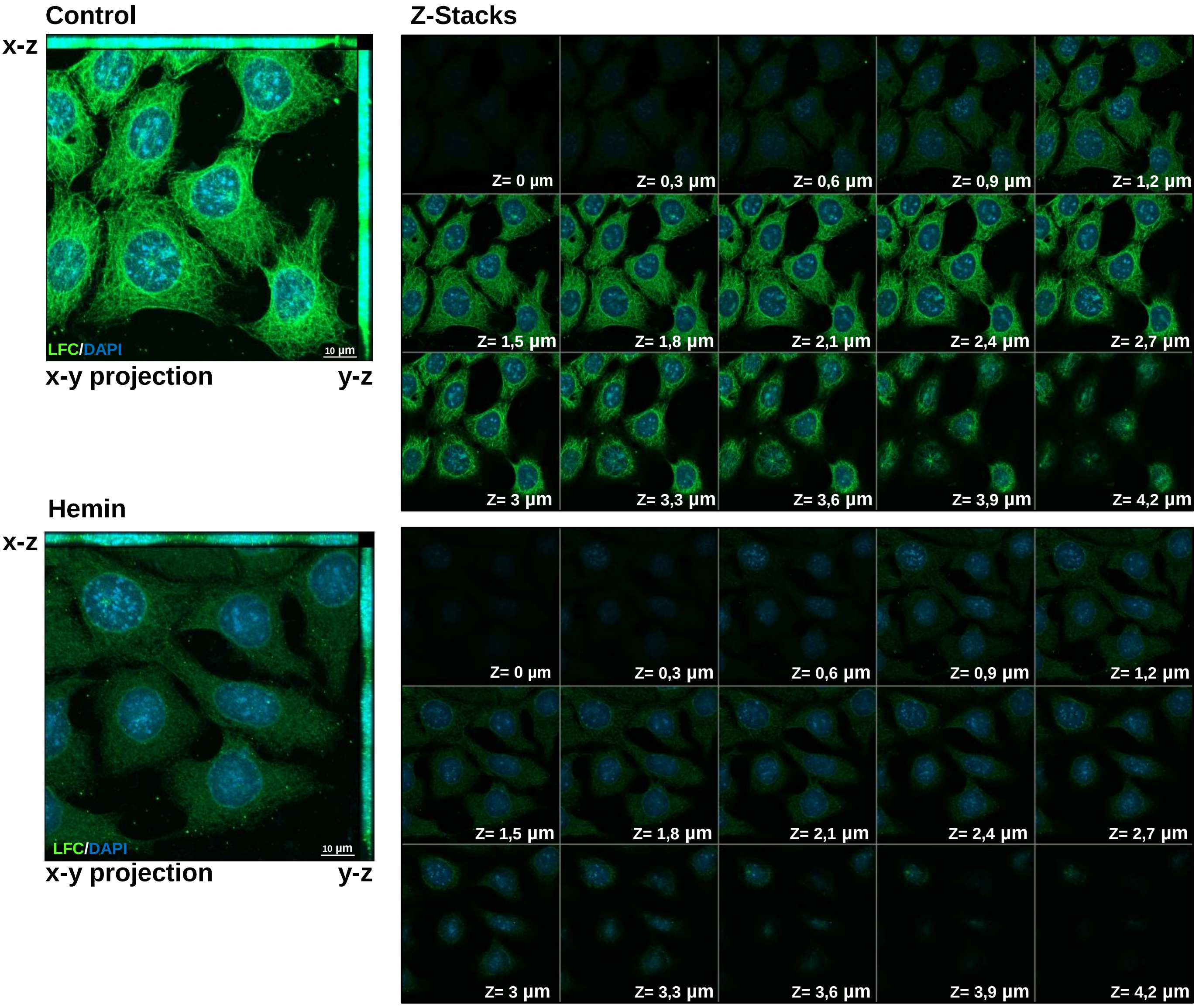

Supplementary Figure 3: Confocal orthogonal view and Z-stacks images of LM3 cells treated with Vehicle (Control) and Hemin for 24h. Z-stack images (total of 15 layers) were taken at 60x magnification with a 0.3 µm slice interval and orthogonal (x/y, x/z or y/z) projection were show of (A) F-actin, (B) Talin, (C) Tubulin and (D) Lfc immunostaining. Nuclei were staining with DAPI (blue). (B) Talin orthogonal projection were performed from Z=0 to Z= 0.9 µm were focal adhesions are localized. The red square represent talin projection from 15 total layers. Scale bar: 10 µm.
