# Supplementary material for: Proteomic analysis of the effect of hemin in breast cancer
Source: Sci Rep. 2023 Jun 21;13:10091. doi: 10.1038/s41598-023-35125-4 (PMC10284804; doi:10.1038/s41598-023-35125-4)
Supplement: Supplementary file 4 — Supplementary Information 4. [file 41598_2023_35125_MOESM4_ESM.pdf]

Supplementary Figure 4

Figure 1B

IB: HO-1

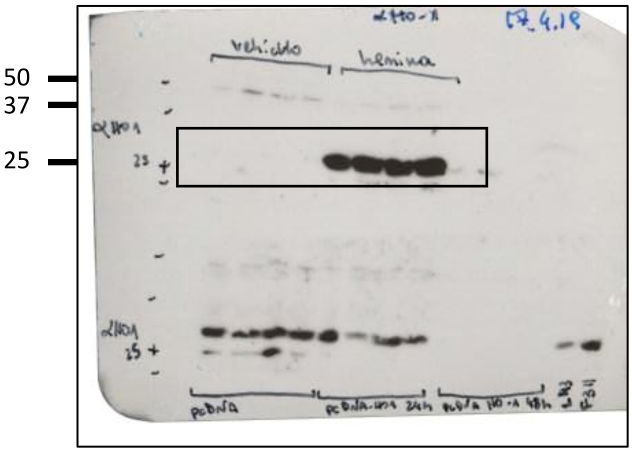

IB: GAPDH

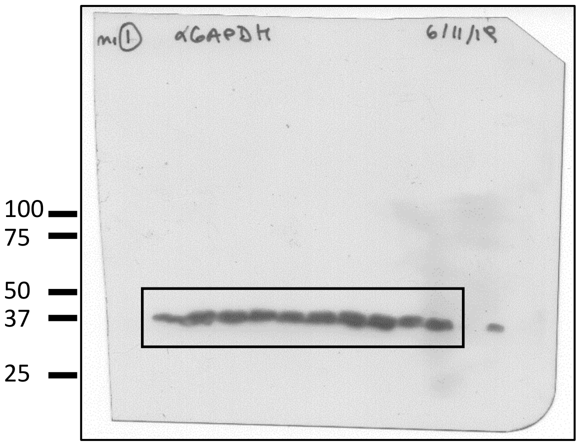

Figure 3B

IB: Smad2/3

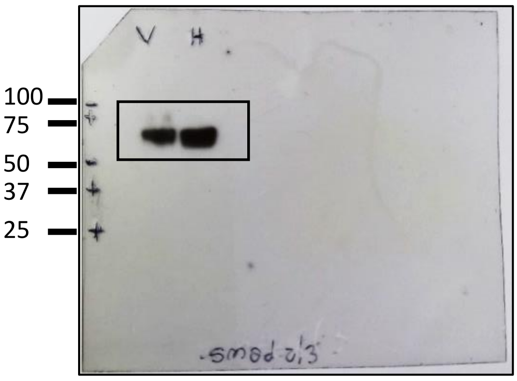

IB: PTEN

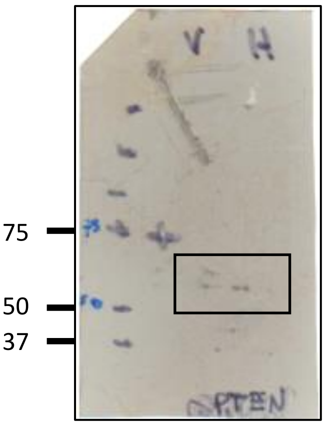

IB: GAPDH

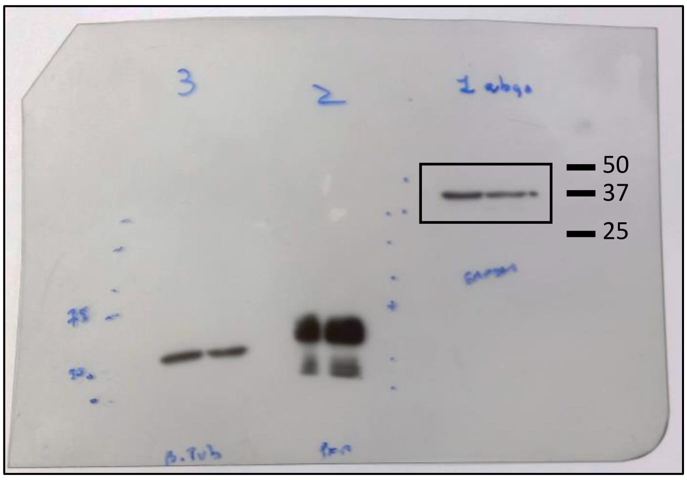

Figure 3C

IB: P53

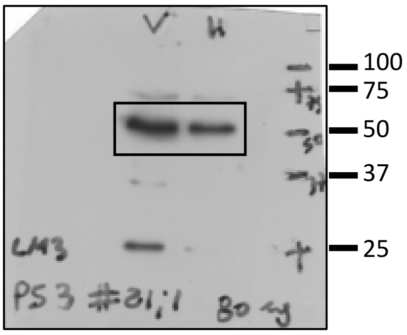

IB: GAPDH

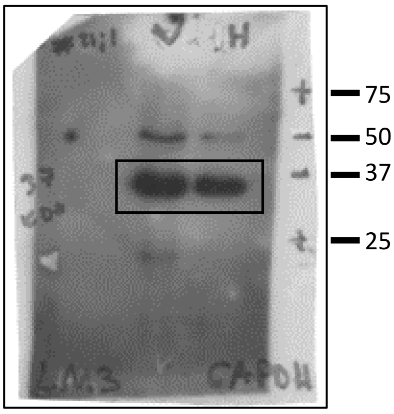

Supplementary Figure 4 continued

Figure 4B

IB: Cathepsin B

IB: GAPDH

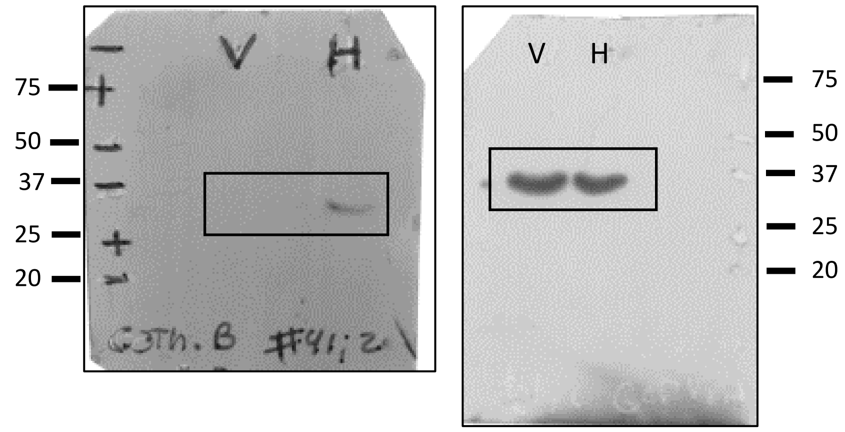

Figure 4C

IB: HO-1

IB: Actin

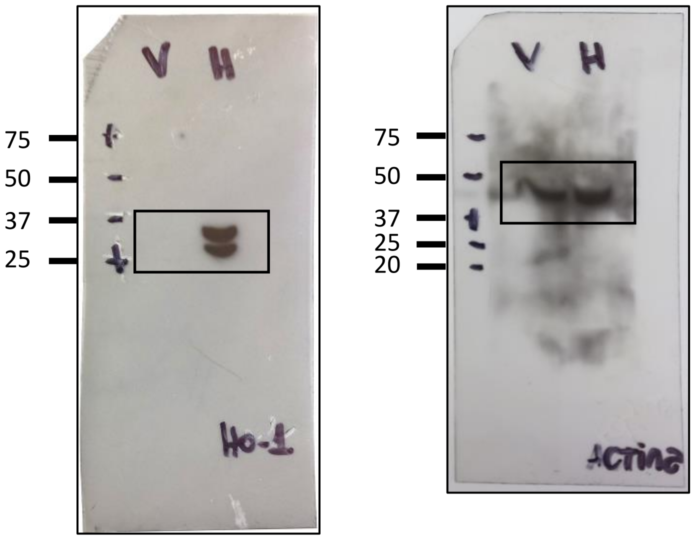

Figure 5D

IB: Talin

IB: GAPDH

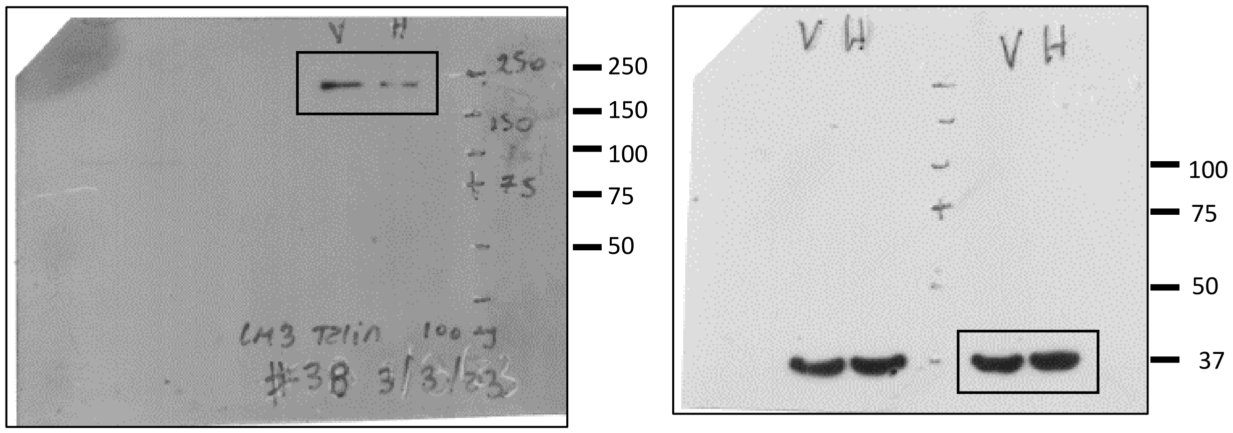

Figure 6E

IB: β-tubulin

IB: GAPDH

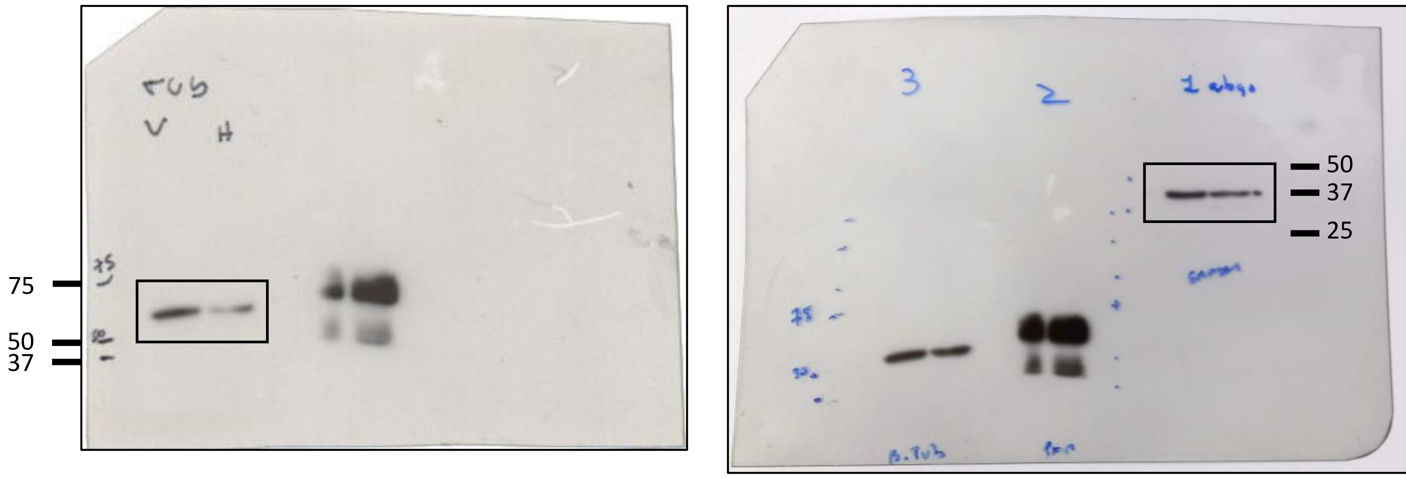

Supplementary Figure 4 continued

Figure 6H

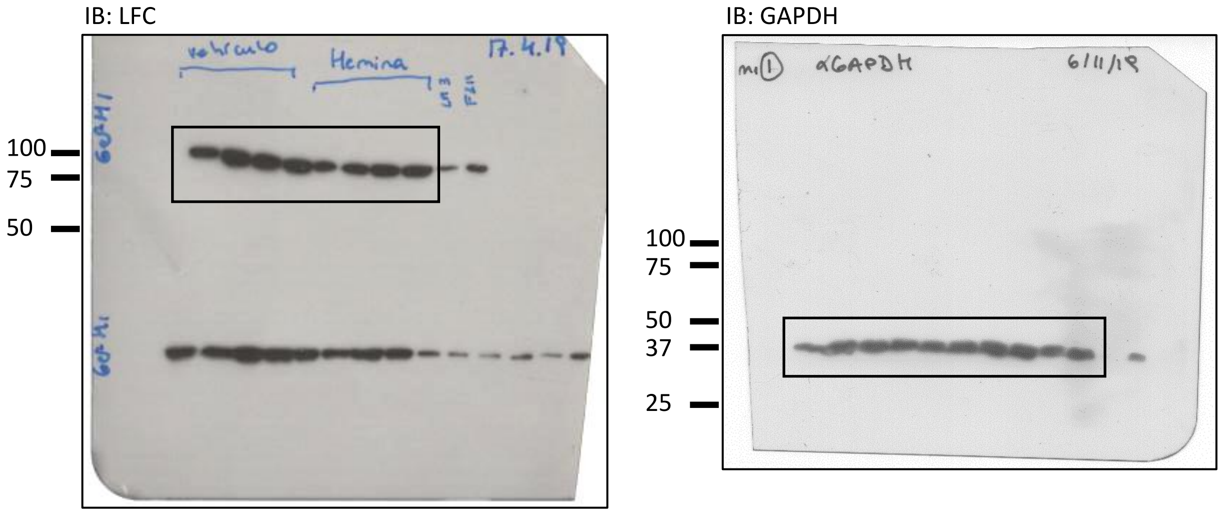

Figure 7C

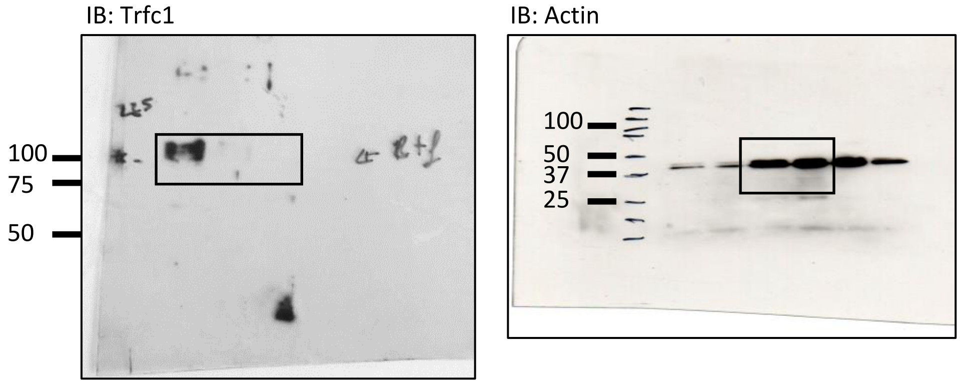

Supplementary Figure 4 : Full unedited gel blots. Black boxes show the cropped bands used in the main figures**.**
